# The lactate sensor GPR81 regulates glycolysis and tumor growth of breast cancer

**DOI:** 10.1038/s41598-022-10143-w

**Published:** 2022-04-15

**Authors:** Shota Ishihara, Kenji Hata, Katsutoshi Hirose, Tatsuo Okui, Satoru Toyosawa, Narikazu Uzawa, Riko Nishimura, Toshiyuki Yoneda

**Affiliations:** 1 Department of Molecular and Cellular Biochemistry, Osaka University Graduate School of Dentistry, 1-8 Yamadaoka, Suita, Osaka 565-0871 Japan; 2 Department of Oral and Maxillofacial Surgery II, Osaka University Graduate School of Dentistry, Osaka, Japan; 3 Department of Oral Pathology, Osaka University Graduate School of Dentistry, Osaka, Japan; 4 Department of Oral and Maxillofacial Surgery, Shimane University Faculty of Medicine, Izumo, Shimane 693-8501 Japan

**Keywords:** Biochemistry, Cell biology, Breast cancer

## Abstract

Metabolic reprogramming is a malignant phenotype of cancer. Cancer cells utilize glycolysis to fuel rapid proliferation even in the presence of oxygen, and elevated glycolysis is coupled to lactate fermentation in the cancer microenvironment. Although lactate has been recognized as a metabolic waste product, it has become evident that lactate functions as not only an energy source but a signaling molecule through the lactate receptor G-protein-coupled receptor 81 (GPR81) under physiological conditions. However, the pathological role of GPR81 in cancer remains unclear. Here, we show that GPR81 regulates the malignant phenotype of breast cancer cell by reprogramming energy metabolism. We found that GPR81 is highly expressed in breast cancer cell lines but not in normal breast epithelial cells. Knockdown of GPR81 decreased breast cancer cell proliferation, and tumor growth. Mechanistically, glycolysis and lactate-dependent ATP production were impaired in GPR81-silenced breast cancer cells. RNA sequencing accompanied by Gene Ontology enrichment analysis further demonstrated a significant decrease in genes associated with cell motility and silencing of GPR81 suppressed cell migration and invasion. Notably, histological examination showed strong expression of GPR81 in clinical samples of human breast cancer. Collectively, our findings suggest that GPR81 is critical for malignancy of breast cancer and may be a potential novel therapeutic target for breast carcinoma.

## Introduction

Cancer cells are characterized by the acquisition of various types of abnormal physiological processes^1^. One of the most fundamental characteristics of cancer is sustained proliferation, which is controlled by growth factors and their corresponding cell surface receptors that act in a paracrine or an autocrine manner. To fuel excessive cell proliferation and tumor growth, cancer cells reprogram energy metabolism using multiple pathways to produce ATP^2,3^. Normal cells mainly use mitochondrial oxidative phosphorylation to produce ATP from glucose under aerobic conditions, whereas ATP production in cancer cells occurs mainly by glycolysis through reprogramming glucose metabolism, even under aerobic conditions^4^. This “aerobic glycolysis” is well established as the Warburg effect and represents one of the anomalous energy-producing metabolic processes observed in cancer^5^. In general, glycolysis produces less ATP than oxidative phosphorylation in mitochondria; however, ATP generation is more rapid, which is suitable for the energy demands of aggressively proliferating cancer cells. An enhanced glycolytic pathway is also advantageous for cancer cells to survive under hypoxic conditions.

Glycolysis is a cytoplasmic metabolic pathway which includes 10 steps of enzymatic reactions, and phosphofructokinase (PFK) represents the rate-limiting step^6^. The final product of glycolysis is pyruvate, which is converted to lactic acid by lactate dehydrogenase-A (LDHA). Thus, metabolic reprogramming from oxidation to glycolysis produces large amounts of lactate in cancer cells^7^. To prevent intracellular acidification, cancer cells release lactate into the tumor microenvironment through monocarboxylic acid transporter 4 (MCT4)^8^. The physiological lactate concentration is approximately 2 mM in healthy tissues, while that in cancer tissues is 10–30 mM^9^. Lactate excretion subsequently creates a low-pH tumor microenvironment, which promotes cancer aggressiveness, including increased angiogenesis, proteolytic activity, metastatic potential, and resistance to anti-cancer therapies^10^.

Lactate has long been recognized as a metabolic waste product of aerobic glycolysis. However, recent studies revealed the critical role of lactate as an energy source in various tissues, including brain, skeletal muscle, and cancer^11–15^. For instance, hypoxic cancer cells produce large amounts of lactate, which is released into the extracellular space via MCT4, and aerobic cancer cells incorporate lactate via MCT1, which is then converted to pyruvate by lactate dehydrogenase B^16^. Pyruvate is used as a substrate for oxidative phosphorylation, and, thus, aerobic cancer cells can utilize lactate as an energy source^16^. This cell–cell exchange of lactate is known as the “lactate shuttle” and allows “metabolic symbiosis” between hypoxic and aerobic cancer cells. Moreover, cancer cells utilize lactate released from cancer-associated stromal cells^17,18^. These reports indicate the important roles of lactate as an energy source for cancer cells.

In addition to its role as an energy source, it has become evident that lactate functions as a signaling molecule by binding to a specific receptor followed by signal transduction. Recently, G protein-coupled receptor 81 (GPR81), also known as hydroxycarboxylic acid receptor 1 (HCAR1), was identified as a lactate receptor in adipocytes^19^. GPR81 is exclusively expressed in adipocytes with relatively low expression in other tissues, including the brain, skeletal muscle, kidney, liver, and in macrophages^19–23^. GPR81 contains seven transmembrane domains, and lactate directly binds to GPR81 resulting in the propagation of intracellular signals through the G protein^24^. The activation of GPR81 by lactate causes cyclic AMP downregulation leading to a decrease in intracellular cAMP concentration^23,25^. The identification of the lactate sensor GPR81 and its downstream signaling highlighted the role of lactate as a signaling molecule, which regulates cellular functions under physiological and pathological conditions. For instance, lactate-dependent GPR81 activation in dendritic cells controls immune evasion of breast cancer cells in a paracrine manner^26^.

Given that the cancer microenvironment contains large amounts of lactate, the activation of GPR81 by lactate likely controls cancer cell activity. However, the pathological role of GPR81 in cancer cells under conditions of high lactate concentrations within the tumor microenvironment remains to be elucidated. In this study, we found that GPR81 is highly expressed in breast cancer cells, and silencing GPR81 expression inhibited proliferation and migration of breast cancer cells in vitro and tumor growth in vivo. Glycolysis and ATP production were also decreased in GPR81-silenced cells. Consistent with these results, human breast cancer tissue specimens demonstrated high GPR81 expression compared with that of normal breast tissues. Our findings suggest that activation of GPR81 by lactate is important for aggressiveness of breast cancer, and GPR81 may be a potential novel therapeutic target for breast cancer.

## Results

### GPR81 is expressed in breast cancer cells

**Figure 1:**
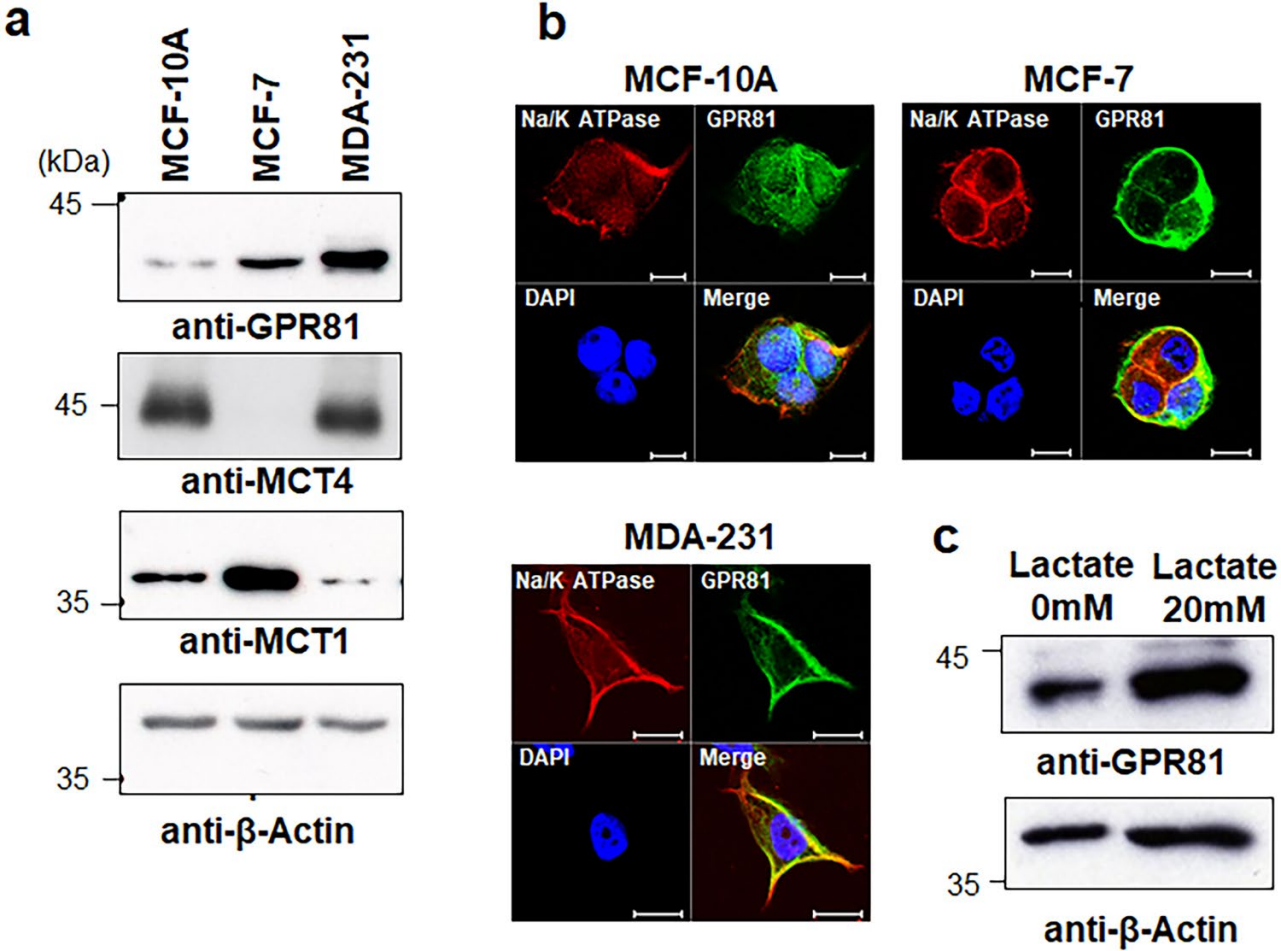
Expression of GPR81 in breast cancer cells. (**a**) Western blot analysis of GPR81 in breast cancer cells (MCF-7 and MDA-MB-231) and non-tumorigenic epithelial cells (MCF-10A). Cell lysates were analyzed by immunoblotting using anti-GPR81, anti-MCT4, anti-MCT1, and anti-β-actin antibodies. (**b**) Immunocytochemical analysis of GPR81 expression in breast cancer cells (MCF-7 and MDA-MB-231) and non-tumorigenic epithelial cells (MCF-10A). MCF-10A, MCF-7 and MDA-MB-231 cells were incubated with polyclonal antibodies against GPR81 followed by Alexa Fluor Plus 488-conjugated secondary antibody and visualized under a fluorescence microscope. Na/K ATPase was used as a cell membrane marker and the nuclei were stained with DAPI. Scale bar 10 μm. (**c**) MDA-MB-231 cells were cultured in the presence and absence of 20 mM lactate for 48 h, and cell lysates were analyzed by immunoblotting using anti-GPR81 and anti-β-actin antibodies.

To determine the pathophysiological roles of GPR81 in breast cancer cells, we first compared the expression level of GPR81 in the tumorigenic human breast cancer cell lines MCF7 and MDA-MB-231 with that in the non-tumorigenic breast epithelial cell line MCF-10A^27^. Western blotting analysis demonstrated that MCF-7 and MDA-MB-231 cells had higher levels of GPR81 protein than MCF-10A cells (Fig. 1a). Immunofluorescent analysis revealed that GPR81 was expressed on the cell membrane of MCF-7 and MDA-MB-231 cells and co-expressed with the cell membrane marker Na/K ATPase (Fig. 1b). In contrast, GPR81 expression was not detectable on the cell membrane of MCF-10A cells (Fig. 1b). The expression of the lactate transporter MCT4 was increased in MCF-10A and MDA-MB-231 cells compared with that in MCF-7 cells (Fig. 1a). In contrast, the expression of MCT1 was highest in MCF-7 cells among these three cell lines (Fig. 1a). Interestingly, lactate itself upregulated the expression of GPR81 in MDA-MB-231 cells (Fig. 1c). These results show that GPR81 expression is increased in breast cancer cells and, thus, suggest that GPR81 plays a role in the regulation of lactate metabolism and is associated with aggressiveness of breast cancer.

### Knockdown of GPR81 suppressed lactate secretion and tumor growth

**Figure 2:**
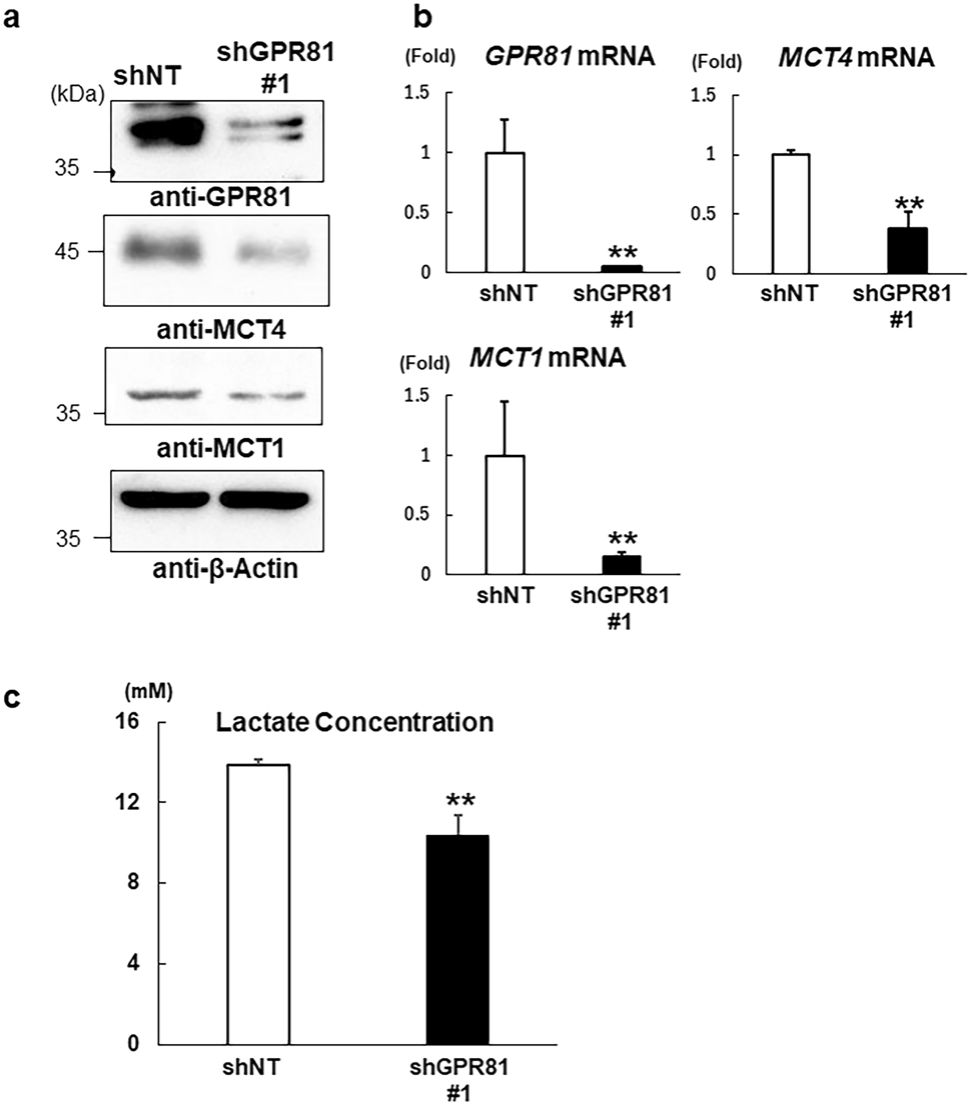
Stable knockdown of GPR81 in MDA-MB-231 cells. Using a lentiviral system, GPR81 was stably knocked down in MDA-MB-231 cells with GPR81-specific short hairpin RNA (shGPR81 #1) and compared with cells containing scrambled shRNA as a control (shNT). Knockdown of GPR81 was confirmed by (**a**) western blotting and (**b**) RT-qPCR. The *GPR81*, *MCT1*, and *MCT4* mRNA levels in the shGPR81 #1 cells are presented as the fold increase compared with those in shNT cells. Data are presented as the means ± s.d. (*n* = 3/group). **p < 0.01; Student’s *t-*test. (**c**) Lactate production in shNT and shGPR81 #1 cells. The cells were cultured for 24 h and lactate concentrations in the supernatants were determined (*n* = 3/group). **p < 0.01 (vs. shNT cells); Student’s *t-*test.

To further determine the role of GPR81 in breast cancer, GPR81 was stably silenced using short hairpin (sh)RNA in MDA-MB-231 cells, which showed the highest expression of GPR81 (Fig. 1a) (hereafter designated shGPR81 cells). We established two MDA-MB-231 lines in which GPR81 was stably suppressed: shGPR81 #1 and shGPR81 #2. We confirmed by RT-qPCR and western blotting that the expression of GPR81 was significantly reduced in shGPR81 #1 and shGPR81 #2 cells compared with that in cells expressing the control hairpin RNA (shNT) (Fig. 2a,b and Supplementary Fig. S1). Of interest, knockdown of *GPR81* also decreased the expression of MCT4 (Fig. 2a,b and Supplementary Fig. S1). Furthermore, shGPR81 #1 cells showed decreased lactate release into the culture media compared with that of shNT cells (Fig. 2c).

**Figure 3:**
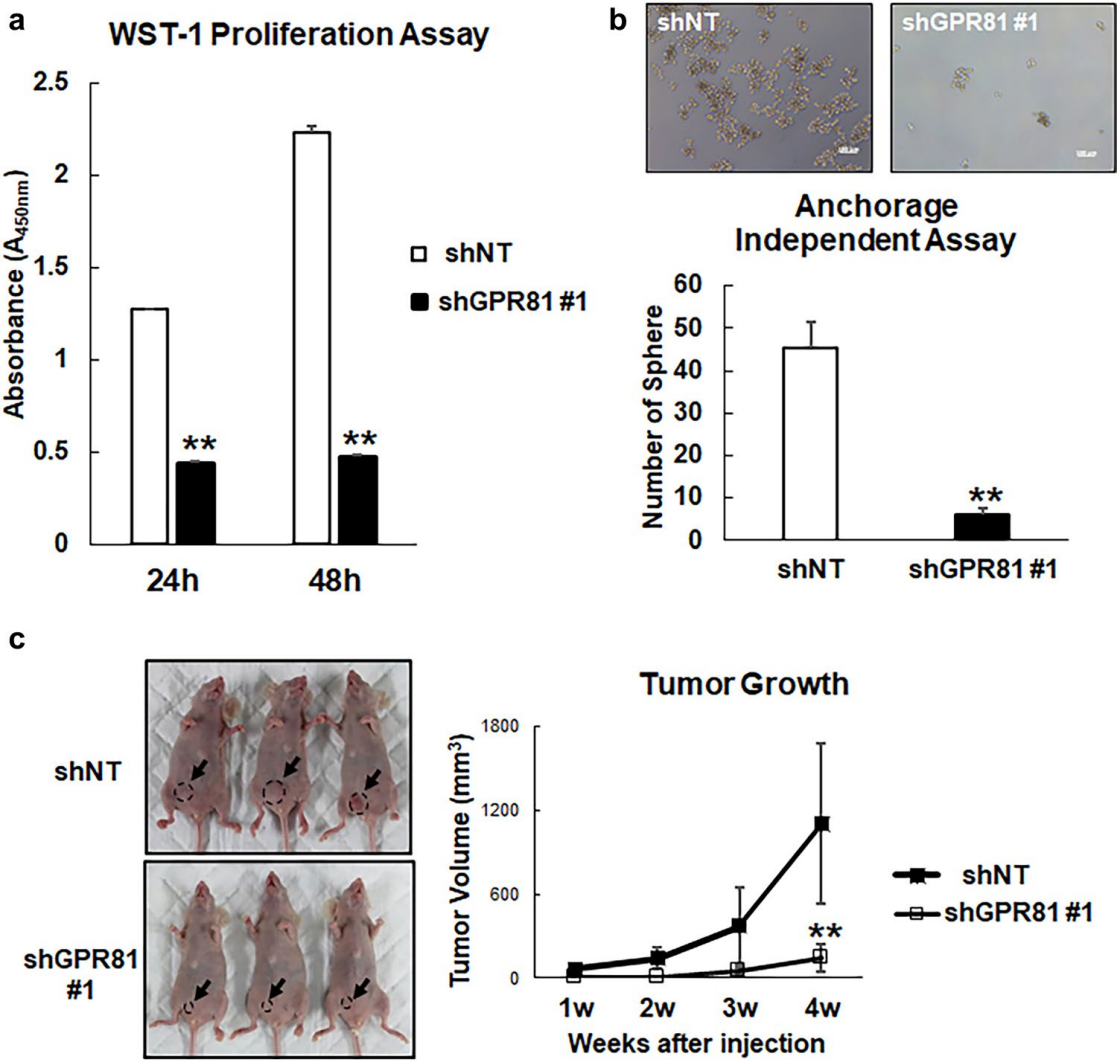
Decrease in cell proliferation and tumor growth of shGPR81 MDA-MB-231 cells. (**a**) ShNT and shGPR81 #1 cells were cultured in 96 well plates, and cell proliferation was determined using the WST-1 Proliferation Assay Kit (*n* = 5/group). **p < 0.01 (vs. shNT cells); Student’s *t*-test. (**b**) Anchorage-independent cell growth of shNT and shGPR81 cells. ShNT and shGPR81 cells were cultured in EZ-BindShut microplates for 7 days, and the number of spheres were counted using a light microscope. The upper panel shows representative micrographs of spheres generated from shNT and shGPR81 cells. Scale bars 100 μm (top). Sphere-forming potential of shNT and shGPR81 cells (mean ± s.d.; *n* = 3). **p < 0.01 (vs. shNT cells); Student’s *t*-test (bottom). (**c**) Representative images of MDA-MB-231 shNT and shGPR81 #1 xenografts at 4 weeks after tumor cell injection (left). Tumor volumes of shNT and shGPR81 #1 xenografts were measured over a period of 4 weeks after cell injection (right; *n* = 5/group). **p < 0.01 (vs. shNT cells); Student’s *t*-test.

Next, we examined the role of GPR81 in cancer cell proliferation. Proliferation of shGPR81 cells was significantly reduced compared with that of shNT cells as assessed by the WST-1 assay (Fig. 3a and Supplementary Fig. S1). Furthermore, anchorage-independent growth of shGPR81 cells was also reduced (Fig. 3b). Importantly, when the shNT or shGPR81 cells were implanted subcutaneously in nude mice, the tumor growth of the shGPR81 cells was substantially decreased compared with that of the shNT cells (Fig. 3c and Supplementary Fig. S1).

**Figure 4:**
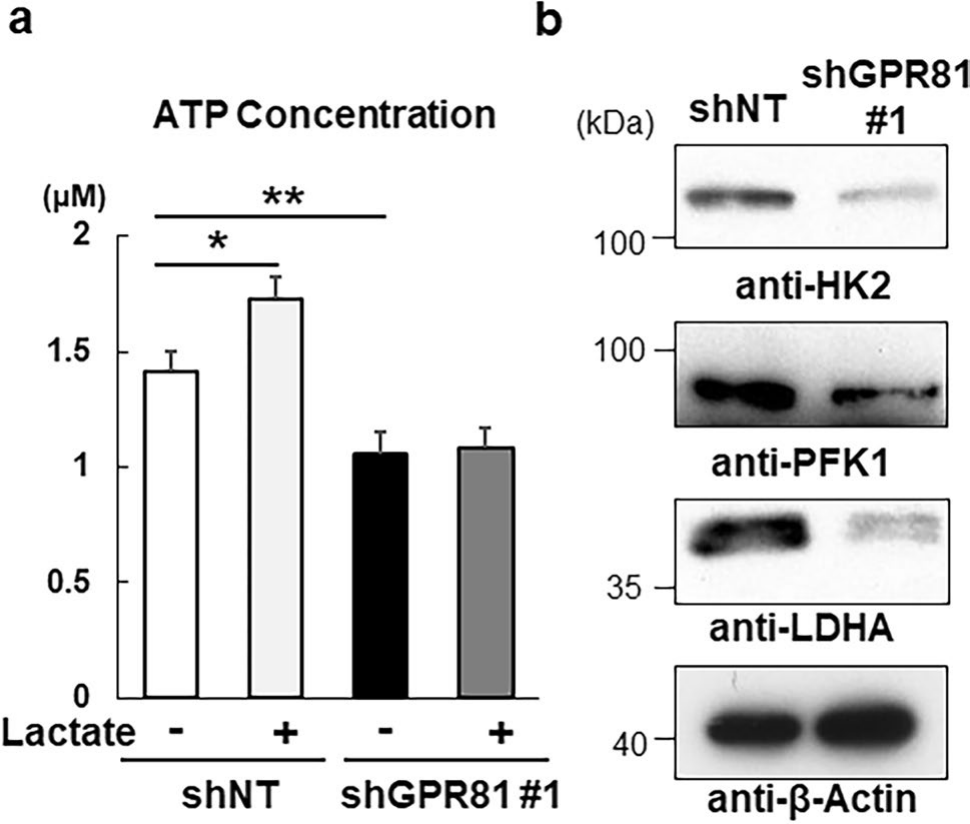
Impaired ATP production and glycolysis in shGPR81 MDA-MB-231 cells. (**a**) ShNT and shGPR81 #1 cells were cultured for 24 h with or without 10 mM lactate and intracellular ATP concentrations were measured using an ATP assay kit (*n* = 3/group). **p < 0.01 using one-way analysis of variance followed by Tukey’s post hoc test. (**b**) Western blot analysis of glycolytic enzymes in shNT and shGPR81 #1 cells. Cell lysates from shNT and shGPR81 #1 cells were analyzed by immunoblotting using anti-HK2, anti-PFK1, anti-LDHA, and anti-β-actin antibodies.

We then examined whether glycolytic ATP production was affected by silencing of GPR81 expression. Lactate treatment increased the ATP production in shNT cells, which was attenuated in shGPR81 cells, suggesting that GPR81 regulated lactate-dependent ATP production (Fig. 4a). Mechanistically, shGPR81 cells showed decreased expression of rate-limiting glycolytic enzymes, including hexokinase 2 (HK2), PFK1, and LDHA, compared with those in shNT cells (Fig. 4b). These data collectively suggest that GPR81 regulates glycolytic ATP production and tumor growth in breast cancer cells. However, it should be noted that reduced expression of the lactate transporters MCT4 and MCT1 may also contribute to decreased ATP production in shGPR81 cells.

### GPR81 is involved in osteolysis associated with tumor growth in bone

**Figure 5:**
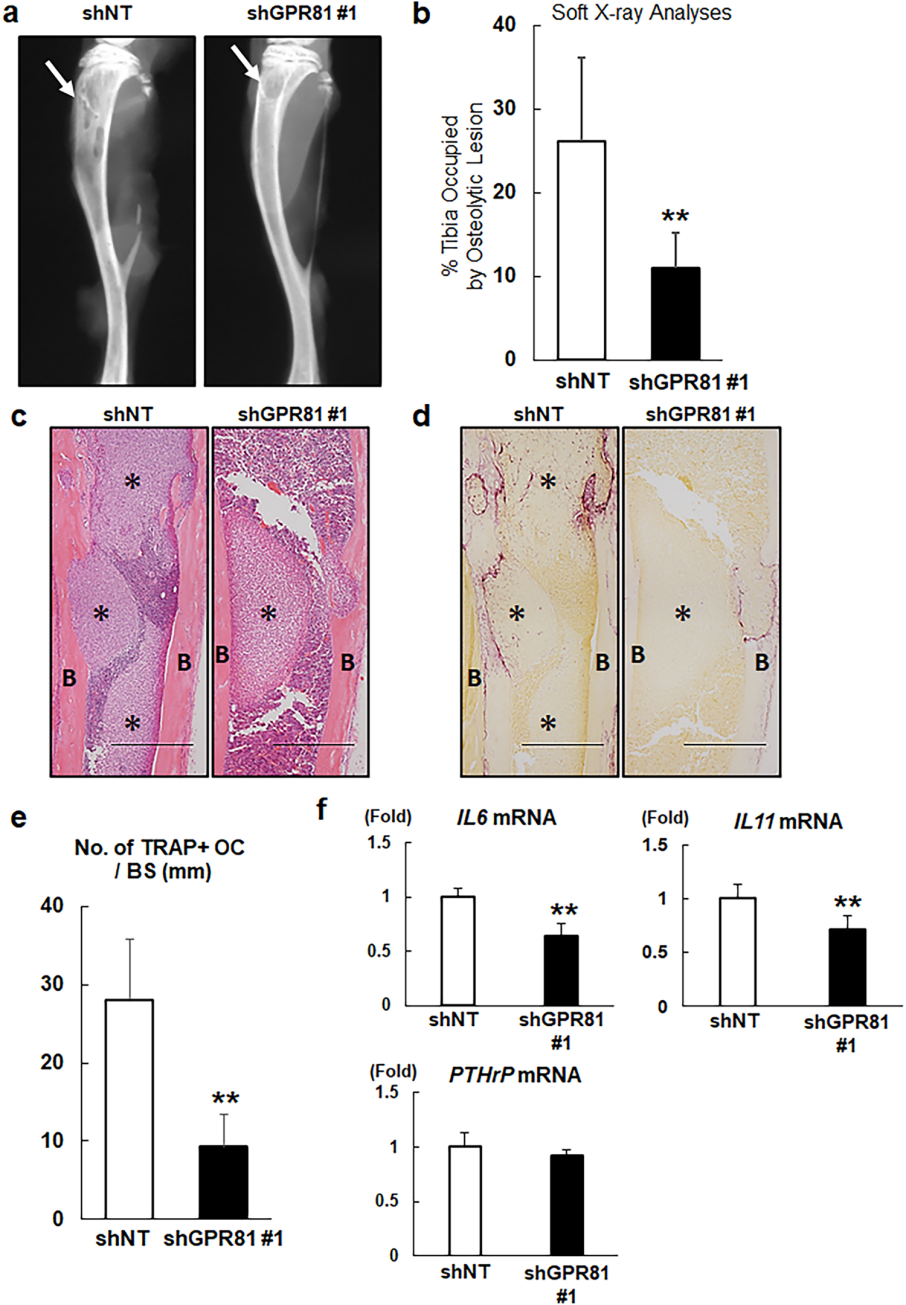
Impaired osteolytic tumor growth of shGPR81-transduced MDA-MB-231 cells. (**a**) Soft X-ray analysis of the hind limbs of mice after bone marrow injections with 1 × 10^5^ shNT or shGPR81 #1 cells. The arrowheads indicate the osteolytic lesions. (**b**) Osteolytic destruction was quantified by measuring the areas of the osteolytic lesions in digital X-ray images. The data are presented as the percentage of the tibia occupied by osteolytic lesions (*n* = 9 mice/group). **p < 0.01 (vs. shNT cells); Student’s *t-*test. (**c**) Hematoxylin and eosin staining of histological sections of the tibiae collected from the mice shown in panel (**a**). B: cortical bone, *: tumor. Scale bars 500 μm (**d**) Tartrate-resistant acid phosphatase (TRAP) staining of serial sections presented in panel (c). Note that TRAP-positive osteoclasts aligned at the endosteal surfaces. B: cortical bone, *: tumor. Scale bars 500 μm (**e**) The number of TRAP-positive osteoclasts per bone surface (no. of TRAP + OC/BS) was measured using a light microscope (mean ± s.d., *n* = 5/group). **p < 0.01 (vs. shNT); Student’s *t*-test. (**f**) Total RNA was isolated from shNT and shGPR81 #1 cells, and *IL6*, *IL11*, and *PTHrP* mRNA expression was analyzed by RT-qPCR. Data are shown as fold changes normalized to shNT (mean ± s.d., *n* = 3/group). **p < 0.01 (vs. shNT); Student’s *t-*test.

Breast cancer frequently metastasizes to bone, and MDA-MB-231 cells have been widely used in animal models of osteolytic bone metastasis^28^. Thus, we next determined whether GPR81 plays a role in the regulation of tumor growth in bone. We injected shNT or shGPR81 cells in the hind limbs of female nude mice and evaluated tumor growth by quantifying the osteolytic lesions by X-ray analysis^29^. We found that the development of osteolytic lesions by shGPR81 cells was significantly decreased compared with that of shNT cells (Fig. 5a,b and Supplementary Fig. S2). Histological analysis further demonstrated that GPR81 was strongly expressed in shNT tumors (Supplementary Fig. S3), and the number of tartrate-resistant acid phosphatase (TRAP)-positive, multinucleated osteoclasts at the tumor-bone interface was significantly decreased in shGPR81 cell-injected bone compared with that of shNT cell-injected bone (Fig. 5c–e). Moreover, we found that knockdown of *GPR81* decreased the expression of interleukin-6 *(IL6)* and *IL11* mRNA*,* whereas no significant change was observed for parathyroid hormone-related protein (*PTHrP*) mRNA expression, contrary to our expectation (Fig. 5f). These data suggest that GPR81 plays an important role in the development of osteolysis and breast tumor growth in bone.

### GPR81 controls cancer cell motility

**Figure 6:**
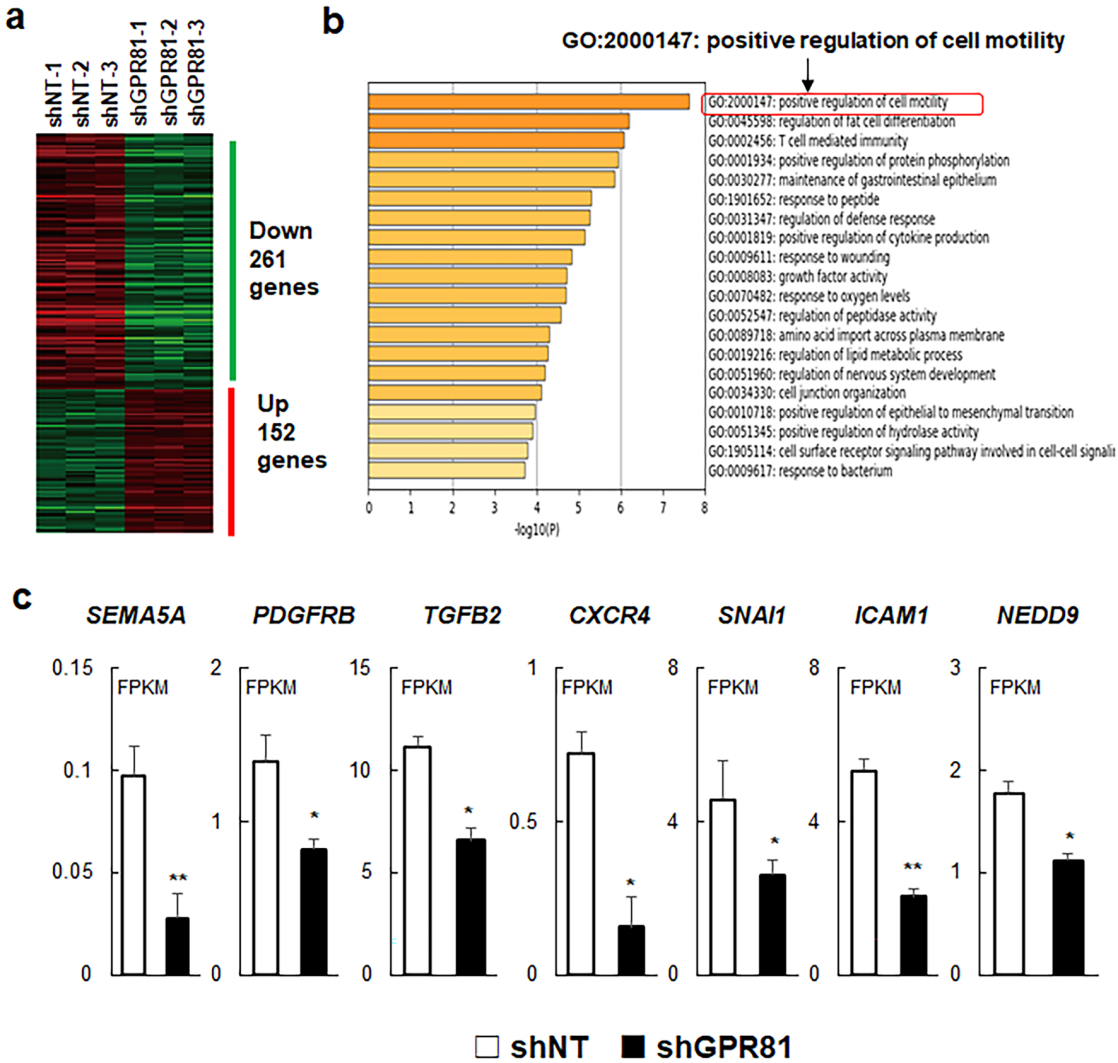
Decreased expression of genes associated with cell motility in shGPR81 cells. (**a**) Heatmap of differentially expressed genes in shNT and shGPR81 cells. Differentially expressed genes (false discovery rate cut off of < 0.05; minimal fold change > 1.5) were identified using the iDEP.94 database. (**b**) Enriched Gene Ontology clusters of downregulated genes. Gene enrichment analysis was performed using the web-based platform Metascape. Note that the positive regulation of cell motility data set showed the greatest enrichment of downregulated genes for shGPR81 cells. (**c**) The mRNA expression of cell motility genes in shNT and shGPR81 cells. The mRNA expression data are presented as fragments per kilobase per million mapped reads (FPKM) (mean ± s.d., *n* = 3/group). **p < 0.01 (vs. shNT); Student’s *t-*test.

To further investigate the role of GPR81 in cancer aggressiveness, we performed comparative global gene expression analysis after RNA-sequencing (RNA-seq) of shNT and shGPR81 cells. RNA-seq analysis identified 261 downregulated and 152 upregulated genes using a threshold for false discovery rate (FDR) of < 0.05 and a fold-change threshold > 1.5 (Fig. 6a). Next, we performed Gene Ontology (GO) enrichment analysis for molecular function and found that shGPR81 cells were significantly enriched for downregulated genes from the positive regulation of cell motility gene set (GO:2000147) (Fig. 6b, Supplementary Table S1). Several genes associated with cancer cell motility, including *SEMA5A, PDGFRB, TGFB2, CXCR4, SNAI1, ICAM1*, and *NEDD9*, were decreased in shGPR81 cells (Fig. 6c).

**Figure 7:**
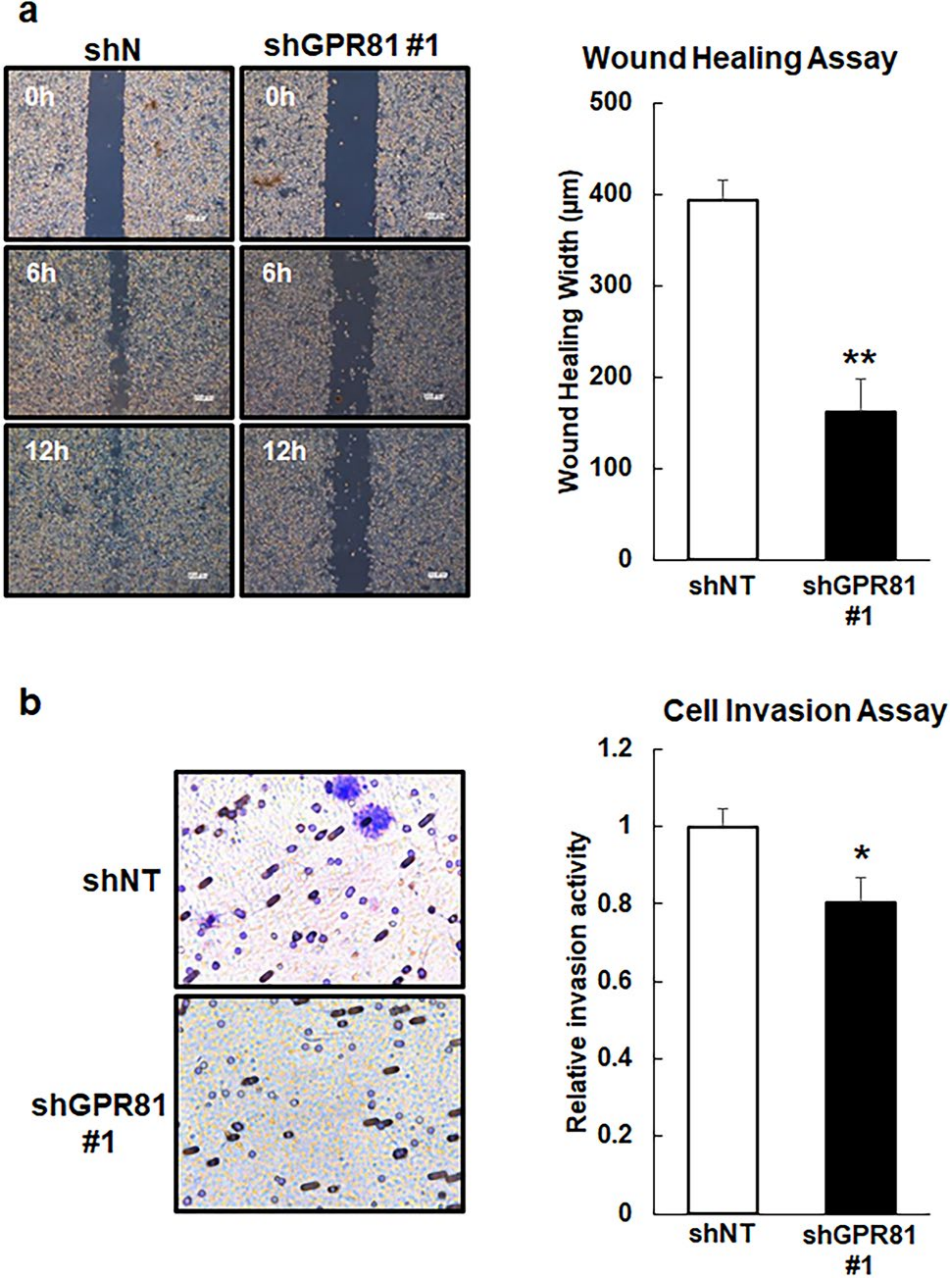
Impaired cell motility and invasion in shGPR81 cells. (**a**) Wound-healing assay for shNT and shGPR81 #1 cells. Monolayers of shNT and shGPR81 #1 cells were scratched, and the distances between the edges of the scratches were measured 12 h after scratching. The data are expressed as the wound healing width that contained migrating cells (*n* = 3/group). **p < 0.01 (vs. shNT); Student’s *t-*test. (**b**) The invasiveness of the cells was evaluated using a Boyden chamber assay. The shNT and shGPR81 #1 cells were seeded into the upper chamber of the apparatus and allowed to grow in serum-free medium. The invading cells were stained with 0.5% crystal violet and counted in 5 random squares using a light microscope (*n* = 3/group). *p < 0.05 (vs. shNT); Student’s *t-*test.

To investigate whether GPR81 regulated cancer cell migration and invasion, we performed wound-healing and cell invasion assays. As expected, the migration and cell invasion activities of the shGPR81 cells were impaired compared with those of shNT cells (Fig. 7a,b and Supplementary Fig. S4). These data suggest that GPR81 contributes to cancer cell invasion and migration.

### GPR81 is highly expressed in clinical samples of human breast cancer

**Figure 8:**
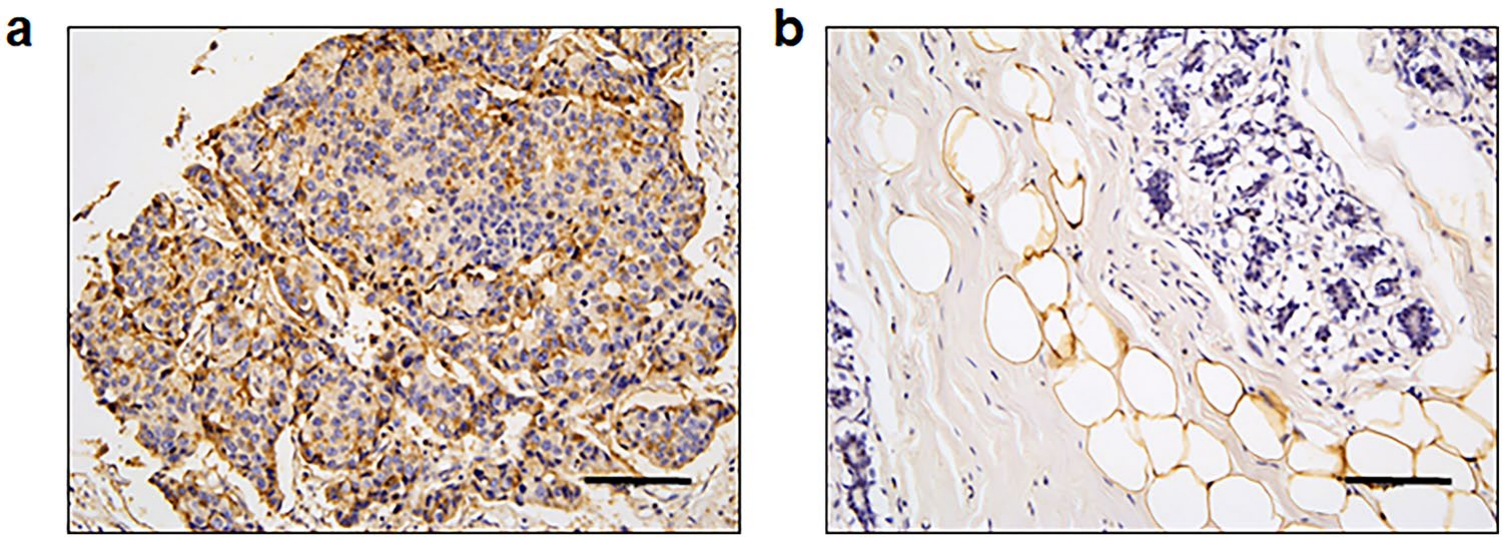
Representative histological views of GPR81 in human breast cancer and normal breast tissue. The expression of GPR81 in human breast cancer (**a**) and adjacent normal breast tissue (**b**) was analyzed by immunohistochemistry using human tissue arrays. Note that GPR81 was expressed in breast cancer tissue and white adipocytes found in normal breast tissue. Scale bar 100 μm.

Finally, we determined the clinical relevance of GPR81 expression in human breast cancers using tissue microarrays. Immunohistochemical analysis showed that 91.7% (22 of 24) of Stage II and 83.3% (5 of 6) of Stage III breast cancers demonstrated positive expression of GPR81 (Fig. 8a, Table 1). GPR81 was not detected in adjacent normal breast tissue, while white adipocytes expressed GPR81 (Fig. 8b). In contrast, stromal cells near the breast cancer tissue showed weak expression of GPR81 (Supplementary Fig. S5). These results suggest that the expression of GPR81 is associated with the degree of malignancy of human breast carcinoma.

**Table 1:** Associations between GPR81 expression and clinicopathological characteristics of human breast cancer specimens (n = 145).

Features	Total no	Sample no	GPR81 expression	
			Negative, n (%)	Positive, n (%)
**TNM**	30			
T2N0M0		17	1 (5.9)	11 (94.1)
T2N1M0		6	1 (16.7)	5 (83.3)
T2N2M0		3	0 (0)	3 (100)
T3N0M0		1	0 (0)	1 (100)
T3N1M0		2	0 (0)	2 (100)
T4N2M0		1	1 (100)	0 (0)
**TNM stage**	30			
Stage I		0	0 (0)	0 (0)
Stage II		24	2 (8.3)	22 (91.7)
Stage III		6	1 (16.7)	5 (83.3)
Stage IV		0	0 (0)	0 (0)

*GPR81* G-protein-coupled receptor 81, *TNM* tumor‑node‑metastasis.

## Discussion

It is well established that cancer cells produce abundant lactate through aerobic glycolysis to fuel sustained tumor growth and secrete lactate into the tumor microenvironment^7^. Despite being exposed to high concentrations of lactate, it remains unclear whether cancer cells sense extracellular lactate and subsequently control their own behavior and energy metabolism. In this study, we demonstrated that activation of GPR81 in breast cancer cells by lactate impacted diverse pathological processes, including aerobic glycolysis, cell proliferation, and cell motility. In addition, histological examination showed rich GPR81 expression in clinical samples of breast tumors. Our findings suggest that GPR81 regulates breast cancer aggressiveness and, thus, is a potential therapeutic target for breast cancer.

We demonstrated that silencing GPR81 inhibited cell proliferation and tumor growth. It is likely that GPR81 is important for cell proliferation by regulating multiple pathways of energy metabolism. Several key enzymes of glycolysis were decreased after silencing GPR81; therefore, GPR81 is involved in the regulation of the glycolytic pathway. Our data also showed that the expression of the lactate transporter MCT4 was decreased in shGPR81 cells. Cancer cells are reported to release and absorb lactate through MCTs and utilize lactate as an energy source under glucose-deficient conditions^16^. GPR81 likely controls lactate transport and subsequent ATP production. Interestingly, recent evidence has shown a critical role for MCTs in tumor development, and MCT1 inhibitors have undergone clinical trials as potential anti-cancer treatments^30,31^. Moreover, Brown et al. reported that tumor-derived lactate activated GPR81 in dendritic cells and, consequently, promoted immune cell evasion by inhibiting tumor-specific antigen presentation^26^. These data collectively suggest that GPR81 signaling in cancer and immune cells is pivotal for tumor growth, and GPR81 may be a promising anti-cancer target. Although the development of antagonists for GPR81 is in the developmental phase thus far, it will be valuable to design and test pharmacological inhibitors of GPR81.

Our results suggest that inhibition of the lactate-GPR81 axis impairs cell motility. It is intriguing that several studies have reported that aerobic glycolysis regulates cell motility^32,33^. Shiraishi et al. reported that aerobic glycolysis is the primary energy source for cancer cell motility, and motility was attenuated by the inhibition of glycolysis but not by the inhibition of mitochondrial ATP production^32^. Moreover, hypoxic conditions promoted cell migratory activity by regulating RhoA-ROCK1 expression^33^. These data demonstrate the interplay between lactate, glycolysis, and cancer cell motility, and our findings suggest GPR81 may be an essential molecule that mediates this interplay^33^. In addition to cell motility, knockdown of GPR81 significantly decreased epithelial–mesenchymal transition-associated genes, including *SNAI1*, *NEDD9*, and *TGFB2*. These data collectively indicate that GPR81 regulates metastatic activity of cancer. Further studies are warranted to determine the role of GPR81 in cancer metastasis.

Breast cancer cells frequently metastasize to bone, and MDA-MB-231 cells have been used to investigate osteolytic bone metastasis of breast cancer *in vivo*^34^. Our data suggested that the knockdown of GPR81 impaired osteolysis and tumor growth partly because of a decrease in the expression of osteolytic cytokines, including IL-6 and IL-11. Both clinical and preclinical studies have reported that IL-6 and IL-11 released from breast cancer cells contributed to the development of bone metastasis^35–37^. Although the molecular mechanism by which GPR81 regulates IL-6 and IL-11 expression remains unclear, it is plausible that lactate controls the production of these cytokines through GPR81 in breast cancer cells. In support of this concept, Gene Ontology analysis of RNA-seq data demonstrated that genes associated with cytokine production (GO:0001819: Positive regulation of cytokine production) were significantly downregulated in shGPR81 cells. Because cytokines are known to regulate diverse processes during tumor progression, it would be of interest to determine the role of GPR81 in cytokine-dependent cancer malignancy.

Recent studies have revealed that lactate contributes to the development of bone metastasis by directly regulating osteoclastogenesis. Qian et al. reported that lactic acid promoted a bone metastatic niche for colorectal cancer^38^. Osteoclasts incorporate lactate that is released from breast cancer cells through MCT1 and use this lactate as a fuel to increase oxidative metabolism, thereby promoting bone resorption^39^. Because our results showed that GPR81 regulated MCT4 expression, these data suggest that GPR81 may indirectly control osteoclast function. Interestingly, it has been reported that lactate controls the inflammatory processes associated with macrophages, which have the potential to differentiate into osteoclasts^40–42^. Expression and function of GPR81 in osteoclasts need to be extensively studied in the future.

Previous studies have established that activation of GPR81 by lactate represses cAMP production through G(i) protein-dependent inhibition of adenylyl cyclase activity^20,22–24^. Importantly, the accumulation of intracellular cAMP has reportedly shown anti-tumor activity in some types of cancer cells. For instance, natural cAMP-elevating compounds, such as forskolin, have been reported to inhibit cell proliferation and migration and induce apoptosis in cancer cells^43–45^. Given that lactate-dependent activation of GPR81 suppressed intracellular cAMP levels, we propose that silencing GPR81 increased the accumulation of cAMP, which resulted in the various types of anti-tumor effects observed in MDA-MB-231 cells. However, it should be noted that cAMP signaling has been reported to both promote and suppress tumor function^46^. Determining correlations between GPR81-dependent intracellular signaling and anti-tumor effects will be an important subject for further investigation.

Although GPR81 is strongly expressed in human breast cancer, the underlying mechanism that controls GPR81 expression in breast cancer remains unclear. Recently, Xie et al. reported that lactate itself promoted GPR81 expression by activating the STAT3 pathway in lung cancer^47^. Lactate also increased GPR81 expression in dendritic cells and promoted immunosuppression^26^. Thus, it would be reasonable to speculate that cancer cells produce and sense lactate through GPR81 to reprogram energy metabolism. However, because lactate mainly accumulates in hypoxic areas, and lactate concentration is not uniform within tumors, other characteristic features of cancer, including low pH, hypoxia, and low nutrients, are likely involved in GPR81 expression. The different expression levels of GPR81 contribute to the heterogeneity of cancer cells. Further analyses to uncover the mechanisms involved in GPR81 expression will be necessary to better understand the role of GPR81 in cancer.

Our results showed that knockdown of GPR81 decreased cancer cell aggressiveness. However, GPR81 knockdown decreased MCT1 and MCT4 expression, and impaired cancer cell aggressiveness observed in shGPR81 cells may have resulted from reduced lactate usage because of decreased MCT expression. Furthermore, pharmacological inhibitors against GPR81 were not tested in this study. A specific GPR81 antagonist that does not affect MCT1 and MCT4 expression will be a useful tool to address mechanistic questions. However, GPR81 antagonists are unavailable at present. Several studies have used 3-hydroxybutyric acid (3-OBA) as an antagonist against GPR81, and 3-OBA treatment showed approximately equivalent effects compared with that of GPR81 knockdown^48,49^; however, there is no experimental evidence that shows specific antagonistic effects of 3-OBA on GPR81^50^. Development of a GPR81-specific antagonist and determining its role in cancer cell activity await further investigation.

In conclusion, our study demonstrates that GPR81 controls tumor activity by regulating lactate transport and glycolytic metabolism in breast cancer cells. We propose that GPR81 may be a novel therapeutic target for breast cancer.

## Materials and methods

### Cell culture

The human breast cancer cell lines MDA-MB-231 and MCF-7 were cultured at 37 °C in a 5% CO_2_ atmosphere in Dulbecco's modified Eagle's medium (DMEM; Nacalai Tesque, Inc.; Kyoto, Japan) supplemented with 10% fetal bovine serum (FBS) and 10,000 units/mL penicillin G, 10,000 μg/mL streptomycin, and 29.2 mg/mL L-glutamine (Wako Pure Chemical Industries, Ltd.; Osaka, Japan). The human mammary epithelial cell line MCF-10A was cultured at 37 °C in a 5% CO_2_ atmosphere in DMEM/Nutrient Mixture F-12 (Thermo Fisher Scientific Inc.; Waltham, MA, USA) supplemented with 100 ng/mL cholera toxin (Sigma-Aldrich; St. Louis, MO, USA), 20 ng/mL epidermal growth factor (PeproTech Inc.; Rocky Hill, NJ, USA), 0.01 mg/mL insulin (Sigma-Aldrich), 500 ng/mL hydrocortisone (Sigma-Aldrich), and 5% horse serum (Thermo Fisher Scientific Inc.).

### Western blotting

The cell lysates were mixed with 4 × Laemmli sample buffer (Bio-Rad) and heated at 95 °C for 5 min. The proteins were separated using SDS-PAGE (7.5%–10% gels) and transferred to nitrocellulose membranes (GE Healthcare). After blocking, the membranes were incubated with primary antibodies overnight at 4 °C and HRP-conjugated secondary antibodies for 1 h. Proteins were visualized with horseradish peroxidase-conjugated anti-mouse or anti-rabbit IgGs using an enhanced chemiluminescence detection kit (Immunostar LD; WAKO; Osaka, Japan). Anti-GPR81 (#NLS2095, 1:1000) was purchased from Novus Biologicals (Littleton, CO, USA). Antibodies against MCT1 (#sc-365501, 1:100) and PFK1 (#sc-166722, 1:100) were purchased from Santa Cruz Biotechnology (Dallas, TX, USA). Anti-MCT4 (#22787-1-AP, 1:10,000) was purchased from Proteintech. Anti-HK2 (#ab104836, 1:1000) was purchased from Abcam (Cambridge, England). Anti-LDHA (#2012, 1:1000) was purchased from Cell Signaling Technology (Danvers, MA, USA), and anti-β-actin (M177-3) was purchased from MBL (Nagoya, Japan). Uncropped images of western blots are shown in Supplementary Fig. S6 online.

### Immunocytochemistry

Cultured cells were washed 3 times with ice-cold phosphate-buffered saline (PBS) and fixed with 3.7% paraformaldehyde in PBS for 20 min. After a 20 min incubation with 0.1% Triton X-100 in PBS, the cells were blocked for 2 h with PBS containing 1% bovine serum albumin, incubated with anti-GPR81 polyclonal antibodies (Novus Biologicals), diluted in 1% bovine serum albumin-PBS, washed 6 times with PBS, and visualized with Alexa Fluor Plus 488-conjugated goat anti-rabbit IgG (H + L) (Thermo Fisher Scientific Inc.). Anti-Na/K ATPase antibody (#ab197713, Abcam) was used to detect this cell membrane marker. Fluorescent images were obtained using a confocal microscope (Leica Microsystems; Wetzlar, Germany).

### Stable knockdown of GPR81

The MDA-MB-231 cells were infected with lentiviral particles that carried either a control shRNA (shNT) or GPR81-specific shRNA (MISSION® shRNA #1, TRCN0000367771 target sequence: GTTGCATCAGTGTGGCAAATA; shRNA #2, TRCN0000008942 target sequence: CGTGTCTGCTAGACTCTATTT; Merck) using polybrene (7 μg/mL). The medium was replaced at 24 h after infection, and the cells were cultured in the presence of 1 μg/mL puromycin to select puromycin-resistant clones. Knockdown of GPR81 was verified by RT-qPCR and western blotting.

### Lactate assay

The lactate concentrations in cell culture media were measured using the Lactate Assay Kit-WST (Dojindo Laboratories; Kumamoto, Japan) and a micro-plate reader (Model 550; Nippon Bio-Rad Laboratories; Tokyo, Japan) in accordance with the manufacturers’ instructions.

### RT-qPCR

Total RNA from cells was extracted using the NucleoSpin RNA Plus total RNA isolation system (Macherey–Nagel GmbH & Co.; Düren, Germany). First-strand cDNAs were synthesized using the ReverTra Ace® qPCR RT Master Mix with gDNA Remover (TOYOBO Co., Ltd., Osaka, Japan). Quantitative real-time reverse transcription-PCR analysis was performed using the Standard Taq PCR protocol, SYBR Green PCR protocol, and a 7300 Real-Time PCR system (Applied Biosystems; Branchburg, New Jersey, USA). TaqMan probes used for the amplification were as follows: human *GPR81* (sense 5ʹ-TGAAACCCAAGCAGCCAGGACACTCAAA-3ʹ, antisense 5ʹ-CCCTCCTTTCCCAAATTCTACAAC-3ʹ, and probe 5ʹ-TGCCACACTGATGCAACTCC-3ʹ); human *MCT1* (sense 5ʹ-GGACCCCAGAGGTTCTCCAG-3ʹ, antisense 5ʹ-TGTCATTGAGCCGACCTAAAAGT-3ʹ, and probe 5ʹ-CCAGGAGGACAGGACAGCATTCCACAATG-3ʹ); human *MCT4* (sense 5ʹ-GGCACCCACAAGTTCTCCAG-3ʹ, antisense 5ʹ-CCGCCAGGATGAACACGTAC-3ʹ, and probe 5ʹ-CCTTCGGGAGGCAAACTCCTGGATGC-3ʹ); and human β-actin (sense 5ʹ-TTAATTTCTGAATGGCCCAGGTCT-3ʹ, antisense 5ʹ-ATTGGTCTCAAGTCAGTGTACAGG-3ʹ, and probe 5ʹ-CCTGGCTGCCTCAACACCTCAACCC-3ʹ). SYBR Green primers used for the amplification were as follows: human *IL6* (sense 5ʹ-GACAGCCACTCACCTCTTCA-3ʹ and antisense 5ʹ-TTCACCAGGCAAGTCTCCTC-3ʹ); human *IL11* (sense 5ʹ-TGAAGACTCGGCTGTGACC-3ʹ and antisense 5ʹ-CCTCACGGAAGGACTGTCTC-3ʹ); human *PTHrP* (sense 5ʹ-ACCTCGGAGGTGTCCCCTAAC-3ʹ and antisense 5ʹ-TCAGACCCAAATCGGACGG-3ʹ); and human β-actin (sense 5ʹ-AGCGGGAAATCGTGCGTG-3ʹ and antisense 5ʹ-CAGGGTACATGGTGGTGGTGCC-3ʹ). Expression levels of mRNAs were normalized to that of β-actin.

### Proliferation assay

The cell proliferation assay was performed using the Premix WST-1 Cell Proliferation Assay System (Roche Holding AG, Basel, Switzerland) in accordance with the manufacturer's protocol. Briefly, MDA-MB-231 cells containing shNT or shGPR81 RNA were plated in 24-well plates (40,000 cells/well) and incubated at 37 °C in a 5% CO_2_ atmosphere. On days 1 and 2, cell proliferation reagent was added to each well and incubated for 1 h. The absorbance was measured using the Model 550 micro-plate reader described above.

### Animal experiments

All animals were handled in accordance with the protocol approved by the Animal Committee of Osaka University Graduate School of Dentistry. The animal studies were in compliance with the ARRIVE guidelines. Subcutaneous injection of MDA-MB-231 cells into nude mice was performed as previously described^51^. MDA-MB-231 cells stably expressing shNT or shGPR81 were suspended in 50 μL Corning Matrigel™ matrix (Corning, NY, USA) plus 50 μL DMEM. MDA-MB-231 cells (5 × 10^6^ cells/100 μL) were inoculated subcutaneously into 5-week-old female Balb/c nude mice under general anesthesia. Anesthesia included a mixture of medetomidine (0.3 mg/kg body weight), midazoram (4.0 mg/kg body weight), and butorphanol (5.0 mg/kg body weight). Tumor volumes were determined at weeks 1, 2, 3, and 4 after inoculation according to the following formula: tumor volume (mm^3^) = length (mm) × width^2^ (mm^2^) × 0.5.

For intratibial injection^52^, MDA-MB-231 cells with stably expressed shNT or shGPR81 RNA (1 × 10^5^ cells/10 µL) were injected into the bone marrow cavities of the right tibiae of 4 to 5-week-old female BALB/c nude mice under general anesthesia as described above. Osteolytic tumor growth was evaluated by quantifying the osteolytic lesions by X-ray analysis as previously described^29^.

### ATP assay

Measurements of ATP concentrations in cell culture media were performed using an Intracellular ATP Measuring Kit Ver.2 (TOYO B-Net CO., LTD.; Tokyo, Japan) and a luminometer (GloMax® Navigator Microplate Luminometer; Promega Corp.; Madison, WI, USA) in accordance with the manufacturers’ instructions.

### TRAP staining

Bone sections were stained for TRAP using the Acid Phosphatase, Leukocyte (TRAP) Kit (Sigma-Aldrich) and analyzed for osteoclastic bone destruction using a light microscope (Leica Microsytems). TRAP + osteoclasts were counted at the tumor-bone interface of the endocortical bone using three fields of view per section for each sample as previously reported^53^. Data are expressed as the number of osteoclasts/bone surface (mm).

### RNA sequencing

Total RNA was extracted as described above. Total RNA libraries were prepared using the TruSeq Stranded mRNA Library Prep kit (Illumina; San Diego, CA, USA) in accordance with the manufacturer’s protocol. Sequencing was performed on an Illumina HiSeq 2500 platform in 75-base single-end mode. Illumina Casava 1.8.2 software was used for base-calling. Sequenced reads were mapped to the human reference genome sequence (hg19) using TopHat v2.0.13 in combination with Bowtie 2 v2.2.3 and SAMtools v0.1.19.

RNA-seq data were analyzed using iDEP (integrated Differential Expression and Pathway analysis)^54^. Briefly, read count data from three replicates each of shNT and shGPR81 expressing cells were generated and uploaded to the iDEP website (http://bioinformatics.sdstate.edu/idep/). Differentially expressed genes were identified using the following thresholds: FDR < 0.05 and minimal fold change > 1.5. Gene Ontology enrichment analysis for molecular function was performed using the Metascape^55^ website (https://metascape.org/gp/index.html#/main/step1). The raw data have been deposited in the NCBI Gene Expression Omnibus database (GSE186211).

### Wound-healing assay

For the wound-healing assay, shNT and shGPR81 cells were cultured for 24 h in DMEM containing 10% FBS in 6 well plates. After confirming that a complete monolayer had formed, the monolayers were wounded by scratching a line through the cellular layer with a standard 200-μL plastic pipette tip. Migration was observed throughout the wound area after 24 h using a phase-contrast microscope equipped with a camera, and migration distances were measured using the photographic images as previously described^56^.

### Migration assay

Cell invasion assays were performed using a Cell Invasion Assay kit (Cell Biolabs Inc.; San Diego, CA, USA) in accordance with the manufacturer's instructions. Briefly, MDA-MB-231 cells expressing shNT or shGPR81 RNA were suspended in serum-free media. The cells (1.5 × 10^5^/chamber) were placed in inserts designed for a 24-well plate. Each insert contained a thin layer of extracellular matrix over a polycarbonate membrane (8 µm pore size). Lower compartments were filled with DMEM containing 10% FBS. After incubation for 6 h at 37 °C in a 5% CO_2_ incubator, cells that invaded and migrated through the matrix-containing membrane and reached the lower surface of the invasion chamber were observed using a phase-contrast microscope with an attached camera. The cells that had migrated were counted using photographic images.

### Tissue microarray

GPR81 expression in invasive human breast carcinoma and normal breast tissues was determined using tissue microarrays (BR245b, BR246b, and BR246d; US Biomax, Inc.; Rockville, MD, USA). The antigen was activated by heating in a citric acid solution. For immunohistochemical analysis of tissues, specimens were incubated with anti-GPR81 antibody (1:100, Novus) overnight at 4 °C, followed by treatment with streptavidin–biotin complex (1:100, EnVision + System-HRP Labelled Polymer; Dako Cytomation; Carpinteria, CA, USA) for 60 min. The tissues were visualized using the Liquid DAB + Substrate Chromogen System (Dako).

### Statistical analysis

Randomization and blinding were not performed in the animal studies*.* Data were statistically analyzed by Student’s *t-*test for comparisons between two groups. For more than two groups, we used one-way analysis of variance (ANOVA) or two-way ANOVA followed by Tukey’s post hoc test. *P*-values < 0.05 were considered statistically significant.

### Ethics

Animal experimentation was performed in strict accordance with the guidelines for proper conduct of animal experiments and related activities of Osaka University Graduate School of Dentistry. All animals were handled in accordance with the protocol approved by the Animal Committee of Osaka University Graduate School of Dentistry.

## Supplementary Information

Supplementary Information.

## Data Availability

RNA-seq data have been deposited in the NCBI Gene Expression Omnibus (https://www.ncbi.nlm.nih.gov/geo/) under accession number GSE186211.

## References

[1] Hanahan D, Weinberg RA (2011). Hallmarks of cancer: the next generation. Cell.

[2] Hsu PP, Sabatini DM (2008). Cancer cell metabolism: Warburg and beyond. Cell.

[3] DeBerardinis RJ, Chandel NS (2016). Fundamentals of cancer metabolism. Sci. Adv..

[4] Gatenby RA, Gillies RJ (2004). Why do cancers have high aerobic glycolysis?. Nat. Rev. Cancer.

[5] Warburg, O. On the origin of cancer cells. *Science* **123**, 309–314. 10.1126/science.123.3191.309 (1956). 10.1126/science.123.3191.309 13298683

[6] Fothergill-Gilmore LA, Michels PA (1993). Evolution of glycolysis. Prog. Biophys. Mol. Biol..

[7] Hirschhaeuser F, Sattler UG, Mueller-Klieser W (2011). Lactate: a metabolic key player in cancer. Cancer Res..

[8] Kennedy KM, Dewhirst MW (2010). Tumor metabolism of lactate: The influence and therapeutic potential for MCT and CD147 regulation. Future Oncol..

[9] de la Cruz-López KG, Castro-Muñoz LJ, Reyes-Hernández DO, García-Carrancá A, Manzo-Merino J (2019). Lactate in the regulation of tumor microenvironment and therapeutic approaches. Front. Oncol..

[10] Corbet C, Feron O (2017). Tumour acidosis: From the passenger to the driver's seat. Nat. Rev. Cancer.

[11] Bergman BC, Tsvetkova T, Lowes B, Wolfel EE (2009). Myocardial glucose and lactate metabolism during rest and atrial pacing in humans. J. Physiol..

[12] Brooks GA (2018). The science and translation of lactate shuttle theory. Cell Metab..

[13] Doherty JR, Cleveland JL (2013). Targeting lactate metabolism for cancer therapeutics. J. Clin. Invest..

[14] Pellerin L (1998). Evidence supporting the existence of an activity-dependent astrocyte-neuron lactate shuttle. Dev. Neurosci..

[15] Quistorff B, Secher NH, Van Lieshout JJ (2008). Lactate fuels the human brain during exercise. Faseb J..

[16] Sonveaux P (2008). Targeting lactate-fueled respiration selectively kills hypoxic tumor cells in mice. J. Clin. Invest..

[17] Koukourakis MI, Giatromanolaki A, Harris AL, Sivridis E (2006). Comparison of metabolic pathways between cancer cells and stromal cells in colorectal carcinomas: A metabolic survival role for tumor-associated stroma. Cancer Res..

[18] Whitaker-Menezes D (2011). Evidence for a stromal-epithelial "lactate shuttle" in human tumors: MCT4 is a marker of oxidative stress in cancer-associated fibroblasts. Cell Cycle.

[19] Ahmed K (2010). An autocrine lactate loop mediates insulin-dependent inhibition of lipolysis through GPR81. Cell Metab..

[20] Ge H (2008). Elucidation of signaling and functional activities of an orphan GPCR, GPR81. J. Lipid Res..

[21] Hoque R, Farooq A, Ghani A, Gorelick F, Mehal WZ (2014). Lactate reduces liver and pancreatic injury in Toll-like receptor- and inflammasome-mediated inflammation via GPR81-mediated suppression of innate immunity. Gastroenterology.

[22] Lauritzen KH (2014). Lactate receptor sites link neurotransmission, neurovascular coupling, and brain energy metabolism. Cereb. Cortex.

[23] Liu C (2009). Lactate inhibits lipolysis in fat cells through activation of an orphan G-protein-coupled receptor, GPR81. J. Biol. Chem..

[24] Kuei C (2011). Study of GPR81, the lactate receptor, from distant species identifies residues and motifs critical for GPR81 functions. Mol. Pharmacol..

[25] Cai TQ (2008). Role of GPR81 in lactate-mediated reduction of adipose lipolysis. Biochem. Biophys. Res. Commun..

[26] Brown TP (2020). The lactate receptor GPR81 promotes breast cancer growth via a paracrine mechanism involving antigen-presenting cells in the tumor microenvironment. Oncogene.

[27] Soule HD (1990). Isolation and characterization of a spontaneously immortalized human breast epithelial cell line, MCF-10. Cancer Res..

[28] Yoneda T, Williams PJ, Hiraga T, Niewolna M, Nishimura R (2001). A bone-seeking clone exhibits different biological properties from the MDA-MB-231 parental human breast cancer cells and a brain-seeking clone in vivo and in vitro. J. Bone Miner. Res..

[29] Sasaki A (1995). Bisphosphonate risedronate reduces metastatic human breast cancer burden in bone in nude mice. Can. Res..

[30] Halford SER (2017). A first-in-human first-in-class (FIC) trial of the monocarboxylate transporter 1 (MCT1) inhibitor AZD3965 in patients with advanced solid tumours. J. Clin. Oncol..

[31] Payen VL, Mina E, Van Hée VF, Porporato PE, Sonveaux P (2020). Monocarboxylate transporters in cancer. Mol. Metab..

[32] Shiraishi, T. *et al.* Glycolysis is the primary bioenergetic pathway for cell motility and cytoskeletal remodeling in human prostate and breast cancer cells. *Oncotarget* **6** (2014). 10.18632/oncotarget.2766 PMC4381583 25426557

[33] Gilkes DM (2014). Hypoxia-inducible factors mediate coordinated RhoA-ROCK1 expression and signaling in breast cancer cells. Proc. Natl. Acad. Sci..

[34] Mbalaviele G (1996). E-cadherin expression in human breast cancer cells suppresses the development of osteolytic bone metastases in an experimental metastasis model. Can. Res..

[35] Kang Y (2003). A multigenic program mediating breast cancer metastasis to bone. Cancer Cell.

[36] Solakoglu O (2002). Heterogeneous proliferative potential of occult metastatic cells in bone marrow of patients with solid epithelial tumors. Proc. Natl. Acad. Sci..

[37] Tawara K, Oxford JT, Jorcyk CL (2011). Clinical significance of interleukin (IL)-6 in cancer metastasis to bone: Potential of anti-IL-6 therapies. Cancer Manag. Res..

[38] Qian J (2021). Lactic acid promotes metastatic niche formation in bone metastasis of colorectal cancer. Cell Commun. Signal.

[39] Lemma, S. *et al.* MDA-MB-231 breast cancer cells fuel osteoclast metabolism and activity: A new rationale for the pathogenesis of osteolytic bone metastases. *Biochim. Biophys. Acta Mol. Basis Dis.* **1863**, 3254–3264. 10.1016/j.bbadis.2017.08.030 (2017). 10.1016/j.bbadis.2017.08.030 28866133

[40] Errea A (2016). Lactate inhibits the pro-inflammatory response and metabolic reprogramming in murine macrophages in a GPR81-independent manner. PLoS ONE.

[41] Yang, K. *et al.* Lactate suppresses macrophage pro-inflammatory response to LPS stimulation by inhibition of YAP and NF-κB activation via GPR81-mediated signaling. *Front. Immunol. ***11**. 10.3389/fimmu.2020.587913 (2020). 10.3389/fimmu.2020.587913 PMC7573489 33123172

[42] Zhou, H.-C. *et al.* Lactic acid in macrophage polarization: The significant role in inflammation and cancer. *Int. Rev. Immunol.* 1–15. 10.1080/08830185.2021.1955876 (2021). 10.1080/08830185.2021.1955876 34304685

[43] Bhola NE, Grandis JR (2008). Crosstalk between G-protein-coupled receptors and epidermal growth factor receptor in cancer. Front. Biosci..

[44] Castoria G (2008). Integrating signals between cAMP and MAPK pathways in breast cancer. Front Biosci..

[45] Stork PJ, Schmitt JM (2002). Crosstalk between cAMP and MAP kinase signaling in the regulation of cell proliferation. Trends Cell Biol..

[46] Zhang H, Kong Q, Wang J, Jiang Y, Hua H (2020). Complex roles of cAMP–PKA–CREB signaling in cancer. Exp. Hematol. Oncol..

[47] Xie Q (2020). A lactate-induced Snail/STAT3 pathway drives GPR81 expression in lung cancer cells. Biochim. Biophys. Acta Mol. Basis Dis..

[48] Shen Z (2015). Inhibition of G protein-coupled receptor 81 (GPR81) protects against ischemic brain injury. CNS Neurosci. Ther..

[49] Chen, S. *et al.* Dual blockade of lactate/GPR81 and PD-1/PD-L1 pathways enhances the anti-tumor effects of metformin. *Biomolecules* **11**. 10.3390/biom11091373 (2021). 10.3390/biom11091373 PMC8466555 34572586

[50] Mohammad Nezhady, M. A. & Chemtob, S. 3-OBA is not an antagonist of GPR81. *Front. Pharmacol.* **12**. 10.3389/fphar.2021.803907 (2022). 10.3389/fphar.2021.803907 PMC8762287 35046827

[51] Hiraga T, Williams PJ, Mundy GR, Yoneda T (2001). The bisphosphonate ibandronate promotes apoptosis in MDA-MB-231 human breast cancer cells in bone metastases. Can. Res..

[52] Wright LE (2016). Murine models of breast cancer bone metastasis. Bonekey Rep..

[53] Hiraga T, Ito S, Nakamura H (2013). Cancer stem-like cell marker CD44 promotes bone metastases by enhancing tumorigenicity, cell motility, and hyaluronan production. Cancer Res..

[54] Ge SX, Son EW, Yao R (2018). iDEP: an integrated web application for differential expression and pathway analysis of RNA-Seq data. BMC Bioinformatics.

[55] Zhou Y (2019). Metascape provides a biologist-oriented resource for the analysis of systems-level datasets. Nat. Commun..

[56] Nishisho T (2011). The a3 isoform vacuolar type H^+^-ATPase promotes distant metastasis in the mouse B16 melanoma cells. Mol. Cancer Res..

