# Supplementary material for: The lactate sensor GPR81 regulates glycolysis and tumor growth of breast cancer
Source: Sci Rep. 2022 Apr 15;12:6261. doi: 10.1038/s41598-022-10143-w (PMC9012857; doi:10.1038/s41598-022-10143-w)
Supplement: Supplementary file 1 — Supplementary Information. [file 41598_2022_10143_MOESM1_ESM.pdf]

Supplementary Information

The lactate sensor GPR81 regulates glycolysis and tumor growth of breast cancer

Shota Ishihara[1,2], Kenji Hata[1]*, Katsutoshi Hirose[3], Tatsuo Okui[4], Satoru Toyosawa[3], Narikazu Uzawa[2], Riko Nishimura[1] and Toshiyuki Yoneda[1]*

1 Department of Molecular and Cellular Biochemistry, Osaka University Graduate School of Dentistry, 1-8 Yamadaoka, Suita, Osaka 565-0871, Japan
2 Department of Oral and Maxillofacial Surgery II, Osaka University Graduate School of Dentistry, Osaka, Japan
3 Department of Oral Pathology, Osaka University Graduate School of Dentistry, Osaka, Japan
4 Department of Oral and Maxillofacial Surgery, Shimane University Faculty of Medicine, Izumo, Shimane 693-8501, Japan

*Corresponding Author:

Supplementary Table S1

GroupID	Term	Description	LogP	Log(q-value)	Symbols
1	GO:2000147	positive regulation of cell motility	-7.63	-3.61	AGT,CD40,COL1A1,CTSH,DEFB1,F3,ICAM1,IL1R1,NEDD9,PDGFRB,PGF,PLCG2,RARRES2,SNAI1,TGFB2,CXCR4,SEMA5A,PDPN,CEMIP,VSIR,DDRGK1,SEMA6D,JAG1,COL15A1,DLX3,HPGD,PDE3B,SERPINF1,RORA,SPINT1,ISM1,BMPER,EVL,ITGB4,S100P
2	GO:0045598	regulation of fat cell differentiation	-6.19	-2.58	JAG1,CEBPA,LPL,RARRES2,RORA,TRIB3,CCDC3,TLCD3B,ZBTB7C,FNDC5,INHBB
3	GO:0002456	T cell mediated immunity	-6.07	-2.53	JAG1,CTSH,HLA-B,HLA-DRA,HLA-DRB1,ICAM1,IL1R1,IL7R,CADM1,SERPINB9,TGFB2,ADA,BCL3,CD40,RORA,TMEM98,CSF2,PBX1,PLCG2,VCAM1,DHRS2,IKZF3,VSIR,GPRC5B,INAVA,CEBPA,COL1A1,NEDD9,PDE3B,PTPRA,TGM2,CXCR4,SEMA5A,PTPRU,PDPN,F3,GPCPD1,PDGFRB,THBD,CST7,CRACR2A,CD14
4	GO:0001934	positive regulation of protein phosphorylation	-5.93	-2.46	ADORA1,AGT,CD40,CSF2,HLA-DRB1,ICAM1,INHBB,NEDD9,PDGFRB,PGF,PLCG2,RARRES2,TGFB2,MAGED1,GDF15,LEFTY1,MAPRE3,GPRC5B,GPRC5C,CEMIP,DDRGK1,AFAP1L2,EEF1A2,TRIB3,SLC1A3,TMPRSS3,CEBPA,COL1A1,F3,PTPRA,ADAMTS3,KIF16B,MVB12B,SLC43A2
5	GO:0030277	maintenance of gastrointestinal epithelium	-5.85	-2.44	MUC2,TFF1,TFF2,TFF3,INAVA,ADORA1,ATP2B2,CHRNA1,CTSH,RAB3D,ILDR2,FLG,CXCR4
6	GO:1901652	response to peptide	-5.30	-2.01	JAG1,AGT,CD40,COL1A1,ICAM1,INHBB,LPL,PDE3B,SERPINF1,PTPRA,RARRES2,TFF1,VCAM1,GLP2R,GDF15,INAVA,TRIB3,CTSH,PGF,SERPINB9,SSTR5,DDRGK1,SLC1A3,TGM2,RRAGD,ZCCHC3,ANKRD1,ADA,SLC6A19
7	GO:0031347	regulation of defense response	-5.26	-2.01	ADA,ADORA1,AGT,CEBPA,HLA-B,HLA-DRB1,IL1R1,LPL,SERPINF1,SERPINB9,PLCG2,RORA,TGM2,CST7,CADM1,ABHD12,GPRC5B,APOBEC3G,ZCCHC3,THBD,SEMA5A,SEMA6D,KREMEN1,CSF2
8	GO:0001819	positive regulation of cytokine production	-5.15	-2.01	AGT,BCL3,CD14,CD40,CSF2,F3,IL1R1,LPL,PLCG2,RORA,CADM1,GPRC5B,NLRP2,INAVA,AFAP1L2,ZCCHC3,CEBPA,PDGFRB,PGF,RARRES2,TGM2,THBD,CXCR4,SEMA5A,HLA-B,HLA-DRB1,INHBB,TGFB2,VSIR
9	GO:0009611	response to wounding	-4.83	-1.84	CD40,COL1A1,F2RL2,F3,INHBB,ITGB4,PDGFRB,PLCG2,SLC1A3,TGFB2,THBD,CXCR4,MYL9,PDPN,KREMEN1,RTN4RL2,ANXA8,ADA,ADORA1,FLG
10	GO:0008083	growth factor activity	-4.72	-1.81	JAG1,AGT,CSF2,INHBB,PGF,TFF1,TGFB2,GDF15,LEFTY1,COL1A1,HPGD,NEO1,PDGFRB,SPINT1,VCAM1,ADAMTS3,ANKRD1,C20orf27,KIF16B,CHRDL1,BMPER,ELAPOR2,SEMA5A,SEMA6D,FNDC5,MTRNR2L1
11	GO:0070482	response to oxygen levels	-4.69	-1.81	ADA,ADORA1,COL1A1,ICAM1,PDGFRB,PGF,RORA,TGFB2,VCAM1,CXCR4,ARNT2,PDPN,ANKRD1,LPL,NNMT,SLC1A3,FZD3,SLC12A5,ITGB4,PDE3B,AGT,ATP2B2,INHBB,KCNJ12,SNTA1,TFF2,CST7,MYL9,WASF3,TMEM98,ADD2,IL1R1,RARRES2,SLC1A4,SLC16A2
12	GO:0052547	regulation of peptidase activity	-4.57	-1.70	AGT,CSNK2A1,CTSH,DAPK1,F3,SERPINA5,SERPINF1,SERPINB9,SPINT1,CST7,NLRP2,VSIR,DDRGK1,ANXA8,ANXA8L1,CEBPA,TMEM98,TRIB3,TGFB2
13	GO:0089718	amino acid import across plasma membrane	-4.31	-1.51	AGT,SLC1A3,SLC1A4,SLC16A2,SLC43A2,ADORA1,PDPN,SLC6A19,SLC26A10,SLCO2A1,KCNJ12,SLC12A5,SLC37A1,TTYH2,ATP2B2,ABCG1
14	GO:0019216	regulation of lipid metabolic process	-4.26	-1.49	ADORA1,AGT,EEF1A2,PDE3B,PDGFRB,PLCG2,RARRES2,RORA,SNAI1,ABCG1,TRIB3,CCDC3
15	GO:0051960	regulation of nervous system development	-4.20	-1.45	CRABP2,SERPINF1,SPINT1,CDKL5,TGM2,CXCR4,FZD3,CST7,SEMA5A,WASF3,DAAM2,TMEM98,SEMA6D,ELAPOR2,NEDD9,AGT,CHRNA1,PDGFRB,PTGFRN,TGFB2,ATRN,GDF15,AUTS2,SLC12A5,ICAM1,LZTS3,PDPN,ANKRD1,EVL,KREMEN1,RTN4RL2,CSNK2A1,CDH11,COL15A1,NEO1,SLC1A3,ITGB4
16	GO:0034330	cell junction organization	-4.10	-1.41	ADD2,AGT,CDH11,CHRNA1,GPC4,ITGB4,PTPRA,SNAI1,SNTA1,CDKL5,TGFB2,PPFIBP2,LZTS3,WASF3,ADGRL1,SHANK2,INAVA,LRFN1
17	GO:0010718	positive regulation of epithelial to mesenchymal transition	-3.95	-1.32	JAG1,COL1A1,SNAI1,TGFB2,PDPN,CRABP2,FLG,HLA-DRB1,KRT83,FZD3,ELAPOR2,PDGFRB,HPGD,SEMA5A,SEMA6D,CEBPA,BMPER
18	GO:0051345	positive regulation of hydrolase activity	-3.91	-1.30	AGT,CD40,CTSH,DAPK1,F3,ICAM1,NEDD9,PDGFRB,RASGRF1,CDKL5,TGM2,NLRP2,RALGAPA2,SIPA1L2,VSIR,DDRGK1
19	GO:1905114	cell surface receptor signaling pathway involved in cell-cell signaling	-3.78	-1.25	ADORA1,CHRNA1,COL1A1,CSNK2A1,GPC4,MPP2,PLCG2,FZD3,SEMA5A,PTPRU,DAAM2,GPRC5B,RNF43,NXN,KREMEN1
20	GO:0009617	response to bacterium	-3.71	-1.19	BCL3,CD14,CD40,CSF2,DEFB1,HLA-B,HLA-DRB1,HPGD,ICAM1,IL7R,SERPINB9,PLCG2,RARRES2,THBD,VCAM1,IKZF3,ANKRD1,INAVA,CEBPA,COL1A1,RORA,CCDC3,AGT,SLC1A3,CXCR4,ABHD12,IL1R1,LPL,SERPINF1,SSTR5,DDRGK1,IGFBPL1

Supplementary Figure S1

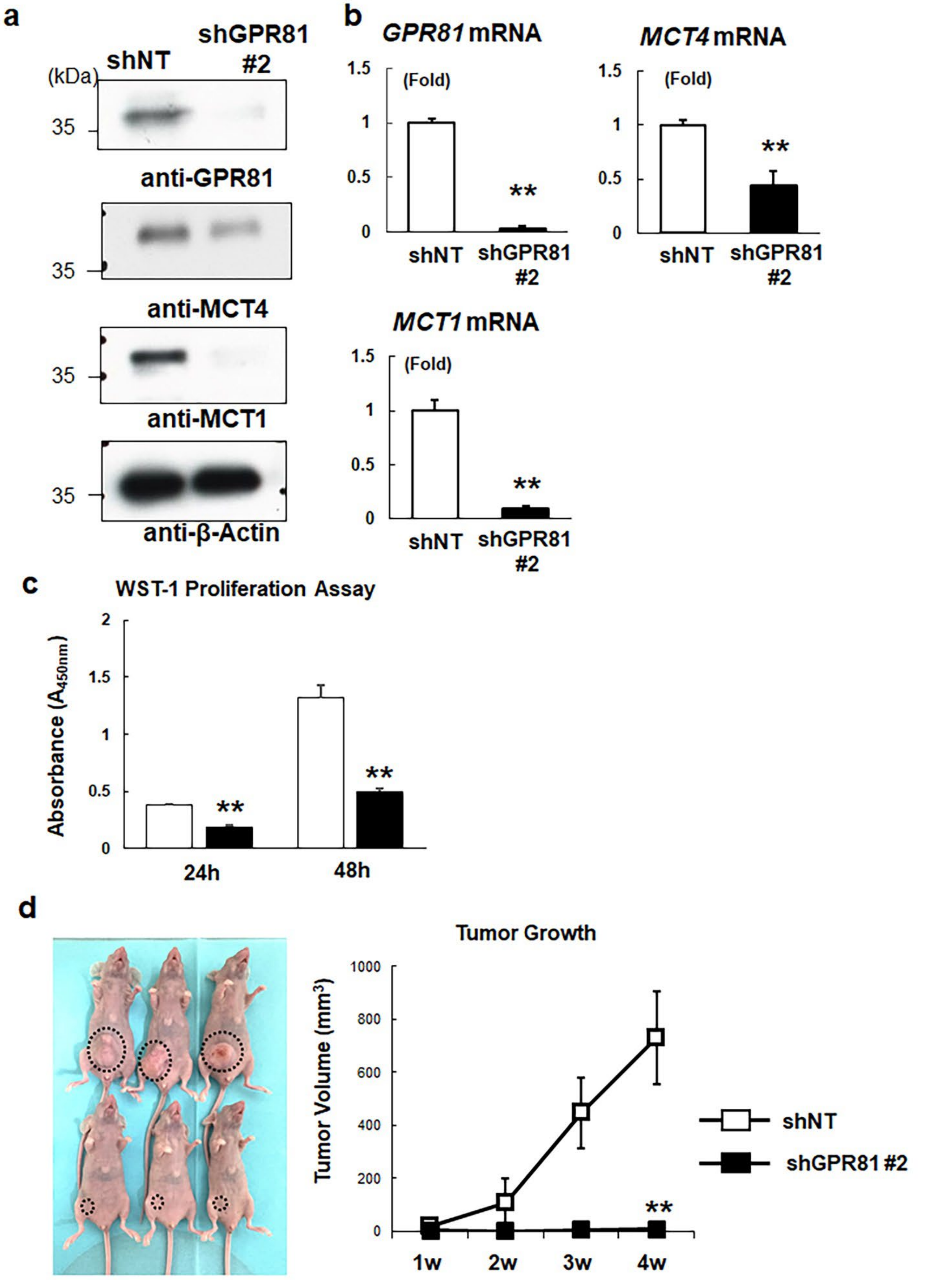

Supplementary Figure S1. Stable knockdown of GPR81 in MDA-MB-231 cells inhibited cell proliferation *in vitro* and *in vivo*. Using a lentiviral system, GPR81 was stably knocked down in MDA-MB-231 cells with short hairpin (sh)GPR81 #2 RNA and compared with cells containing scrambled shRNA as a control (shNT). Knockdown of GPR81 was confirmed by (**a**) western blotting and (**b**) RT-qPCR. The *GPR81*, *MCT1*, and *MCT4* mRNA levels in the shGPR81 #2 cells are presented as the fold increase compared with that of shNT cells. Data are presented as the means ± s.d. (n = 3/group). **$p < 0.01$; Student's t-test. (**c**) ShNT and shGPR81 #2 cells were cultured in 96 well plates, and cell proliferation was measured using the WST-1 Proliferation Assay Kit (n = 5/group). **p < 0.01 (vs. shNT cells); Student's t-test. (**d**) Representative images of MDA-MB-231 shNT and shGPR81 #2 xenografts at 4 weeks after tumor cell injection (left). Tumor volumes of shNT and shGPR81 #2 xenografts were measured over a period of 4 weeks after cell injection (right; n = 5/group). **p < 0.01 (vs. shNT cells); Student's t-test.

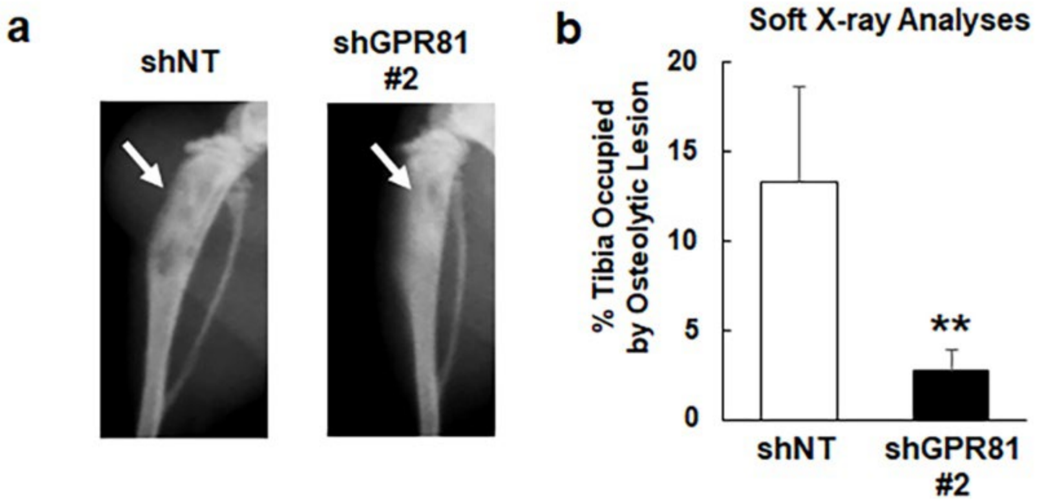

Supplementary Figure S2. Impaired osteolytic tumor growth of shGPR81 #2 MDA-MB-231 cells. (a) Soft X-ray analysis of the hind limbs of mice after bone marrow injections with 1 × 10^5 shNT or shGPR81 #2 cells. The arrowheads indicate the osteolytic lesions. (b) Osteolytic destruction was quantified by measuring the areas of the osteolytic lesions in digital X-ray images. The data are presented as the percentage of the tibia occupied by osteolytic lesions (n = 7 ~ 9 mice/group). **p < 0.01 (vs. shNT cells); Student's t-test.

Supplementary Figure S3

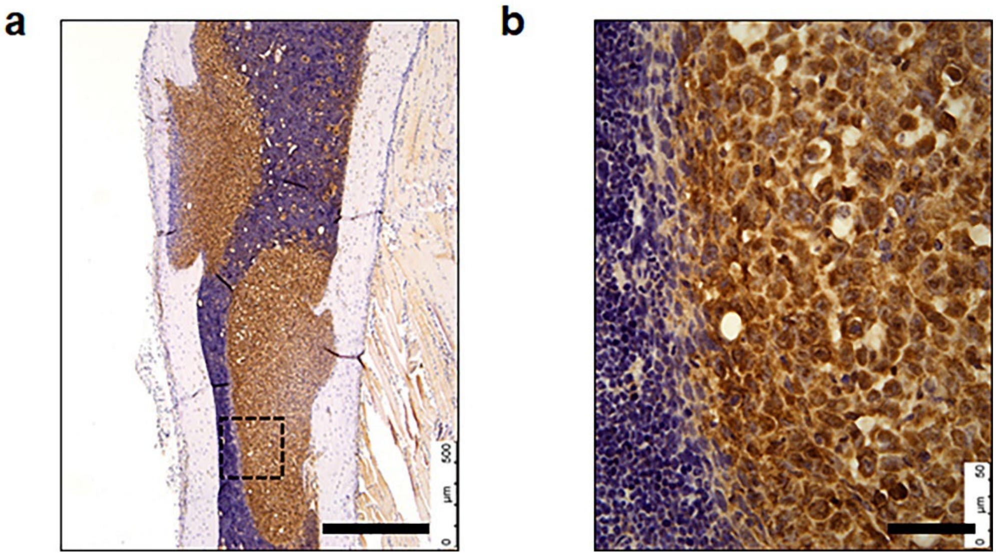

Supplementary Figure S3. The expression of GPR81 in shNT MDA-MB-231 cells (**a**) The expression of GPR81 in hindlimbs injected with 1 × 10^5 shNT MDA-MB-231 cells. Note that GPR81 was strongly expressed in shNT MDA-MB-231 cells in the tibia. Scale bar: 100 µm (**b**) High magnification image of the dashed box in (a). Scale bar: 50 µm.

Supplementary Figure S4

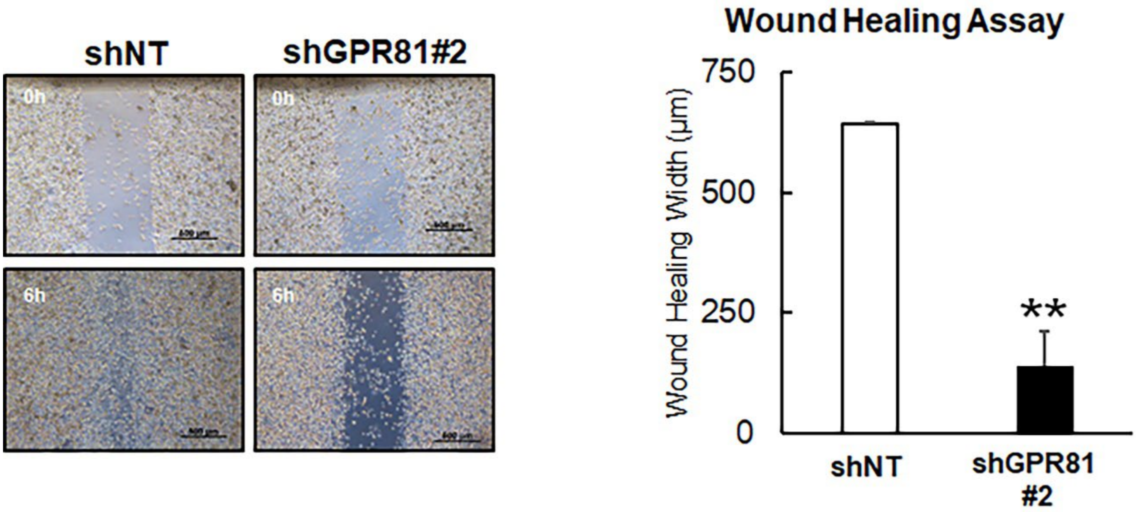

Supplementary Figure S4. Impaired cell motility and invasion by shGPR81 cells. Wound-healing assays for shNT and shGPR81 #2 cells. Monolayers of shNT and shGPR81 #2 cells were scratched, and the distances between the edges of the scratches were measured 12 h after scratching. The data are expressed as the wound healing widths of the original wound that contained migrating cells (n = 3/group). **p < 0.01 (vs. shNT); Student's t-test.

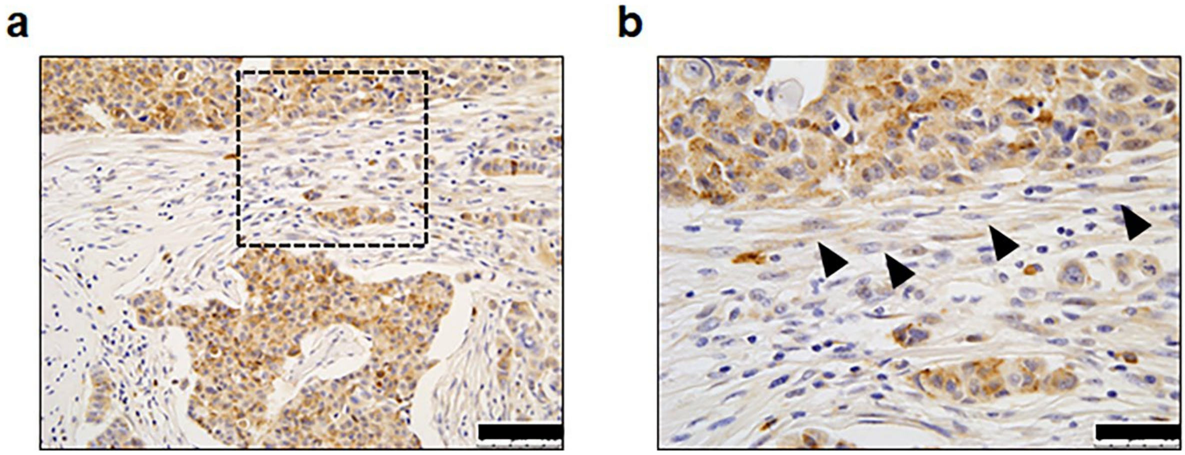

Supplementary Figure S5. The expression of GPR81 in human breast cancer (a) The expression of GPR81 in human breast cancer was determined by immunohistochemistry using human tissue arrays. Scale bar: 100 µm. **(b)** High magnification image of the dashed box in (a). The arrowheads indicate the stromal cells with weak GPR81 expression. Scale bar: 50 µm.

Figure 1 a

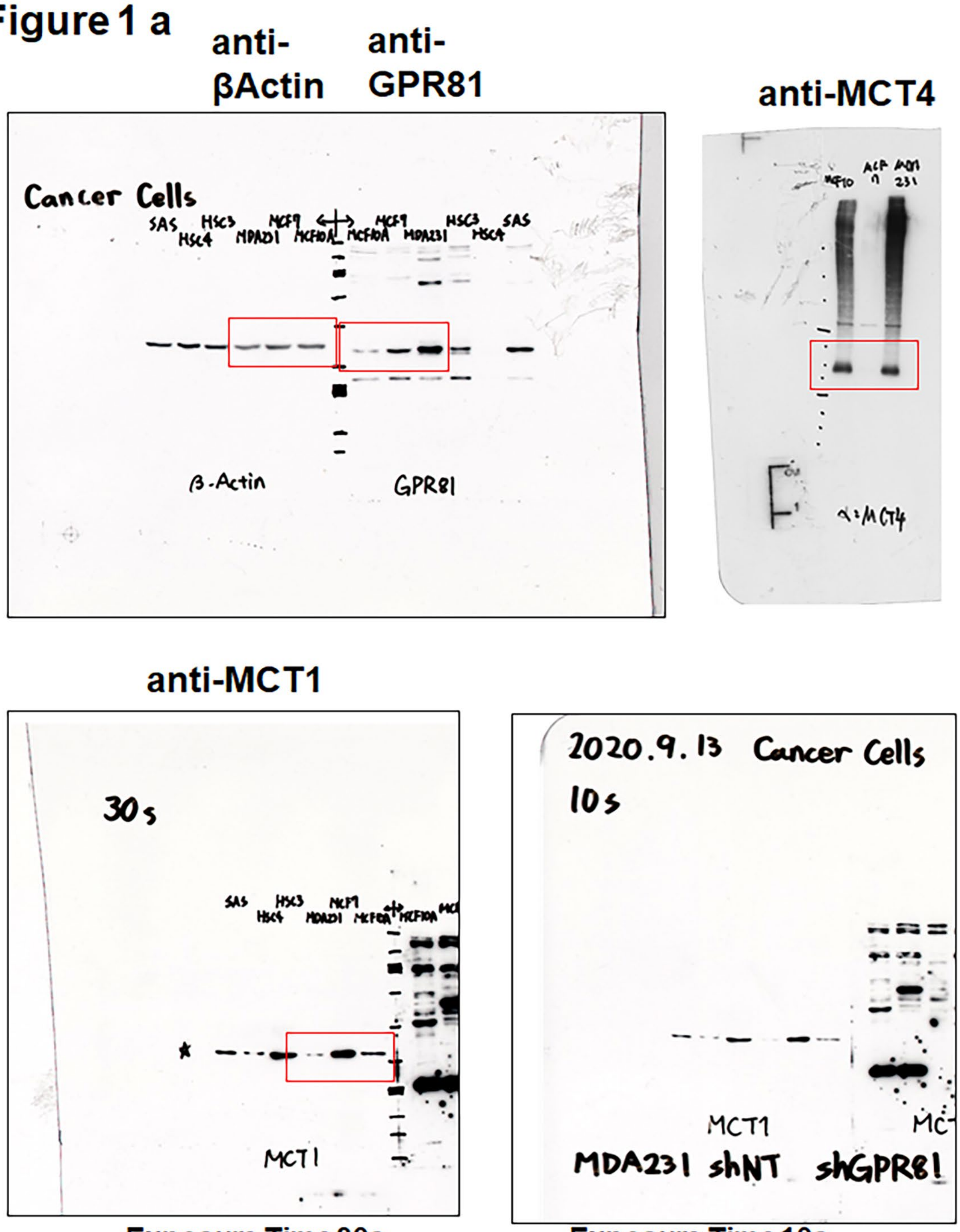

Exposure Time 30s

Exposure Time 10s

Supplementary Figure S6

Figure 2 a

anti-βActin **anti-GPR81**

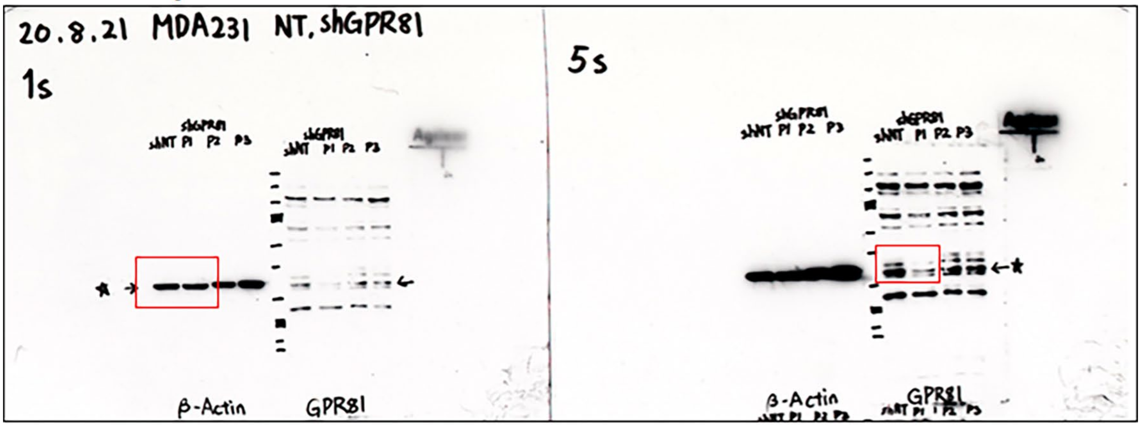

Exposure Time 1s **Exposure Time 5s**

anti-MCT4

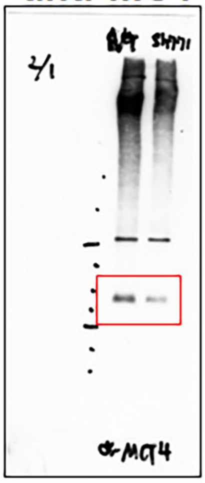

anti-MCT1

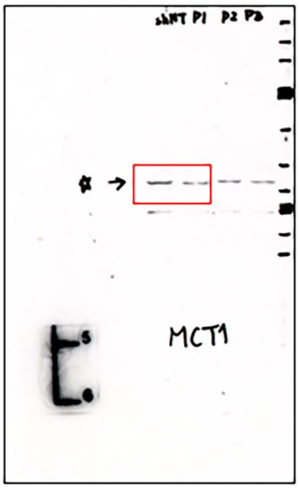

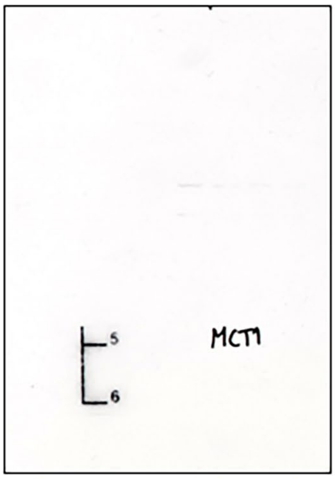

Exposure Time 5s **Exposure Time 1s**

Figure 4b

anti-HK2 anti-LDHA anti-PFK1

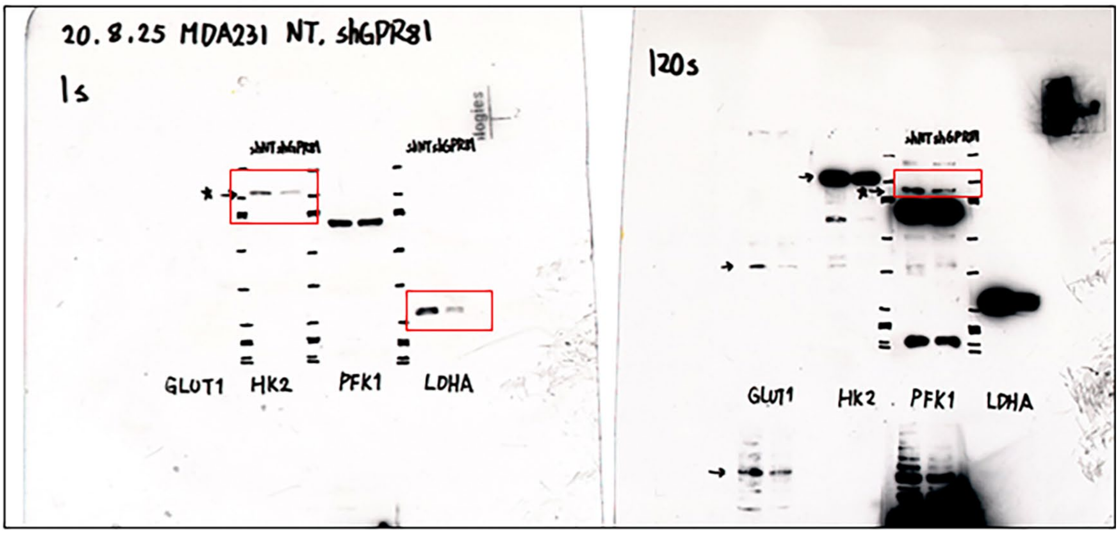

Exposure Time 1s

Exposure Time 120s

anti-βActin

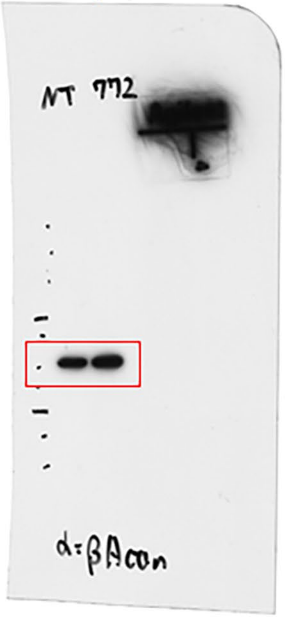

Supplementary Figure S1a

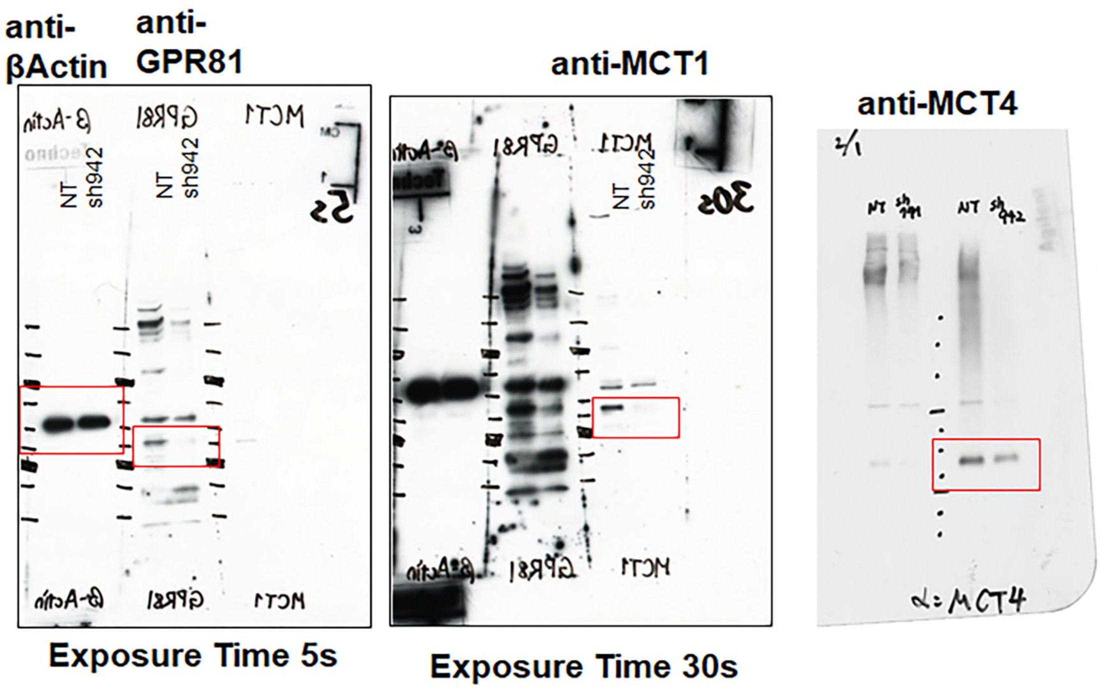

Supplementary Figure S6. Uncropped western blots for Figures 1, 2, 4, and Supplementary Figure S1.The original immunoblots for the main and supplementary figures are shown. The red frames indicate the bands that are shown in the corresponding figures.
